# Seed and peel essential oils obtained from *Campomanesia adamantium* fruit inhibit inflammatory and pain parameters in rodents

**DOI:** 10.1371/journal.pone.0157107

**Published:** 2017-02-21

**Authors:** Danieli Zuntini Viscardi, Jucicléia da Silva Arrigo, Camila de Azevedo Chaves Correia, Cândida Aparecida Leite Kassuya, Claudia Andrea Lima Cardoso, Iriani Rodrigues Maldonade, Eliana Janet Sanjinez Argandoña

**Affiliations:** 1 College of Exact and Technological Sciences, Federal University of Grande Dourados, Dourados, Mato Grosso do Sul, Brazil; 2 College of Health Sciences, Federal University of Grande Dourados, Dourados, Mato Grosso do Sul, Brazil; 3 Course of Chemistry, State University of Mato Grosso do Sul, Dourados, Mato Grosso do Sul, Brazil; 4 Brazilian Agricultural Research Agency (Embrapa), Brasília, Federal District, Brazil; 5 College of Food Engineering, Federal University of Grande Dourados, Dourados, Mato Grosso do Sul, Brazil; University of Pittsburgh, UNITED STATES

## Abstract

*Campomanesia adamantium* (Myrtaceae) is popularly known as “gabiroba” and has been used in folk medicine as antirheumatic, antidiarrheal, hypocholesterolemic and anti-inflammatory. This study evaluated the anti-inflammatory and antinociceptive activities and toxicology of essential oils from peel (EOP) and seed (EOS) of *C*. *adamantium* fruits in animal models. Different groups were treated with doses of 100 and 300 mg/kg and the inflammatory parameters were evaluated in carrageenan induced paw oedema and leukocyte migration in pleurisy model, while antinociceptive activity was evaluated using formalin method in rodents. The major constituent of EOP and EOS was limonene with 13.07% and 20.89%, respectively. No clinical signs of toxicity have been observed in animals. It was observed a significant decreased (*P*<0.01) in leukocyte migration at the dose of 300 mg/kg of EOP and EOS, with maximal inhibition of 89±3% for EOP and 80±6% for EOS. Paw oedema was inhibited at all times, and maximal inhibition was at the dose of 100 mg/kg at 2 h after carrageenan injection with 72±2% for EOP and 74±2% for EOS. EOS and EOP also reduced the first and second phases of formalin-induced nociception test. In the first formalin-phase, maximal inhibitions were at 48±5% for EOP and 66±4% for EOS (300 mg/kg). At the inflammatory phase induced by formalin, maximal inhibitions were 72±2% for EOP and 80±2% for EOS at the dose of 100 mg/kg. Seed and peel essential oils from *C*. *adamantium* fruit inhibited leukocyte migration, inflammatory and neurogenic pain and oedema suggesting their use as nutraceutical or pharmacological agent.

## Introduction

Cerrado region is the second largest bioma in Brazil, after Amazonia. This ecosystem comprises more than 7,000 plant species [1]. Due to its diverse flora, research interest has been increased and endemic medicinal plants from Cerrado have been a source of bioactive compounds. *Gabiroba* or *guabiroba-do-campo* or *guavira*, the fruit of *Campomanesia adamantium* (Cambess.) O. Berg, is widely found and used in areas of Cerrado, mainly in the Midwest region and Atlantic Forest in the Southeast and South regions of Brazil [2].

*C*. *adamantium* belongs to Myrtaceae family and their fruits, as well as fruits from other species of *Campomanesia*, are traditionally used in the production of homemade liqueurs, juices, and sweets [3] and also employed in folk medicine as antirheumatic, antidiarrheal, hypocholesterolemic, anti-inflammatory [4], and for the treatment of cystitis, and urethritis [5].

Our group has previously shown that hydroalcoholic extract from *C*. *adamantium* fruit barks exhibited anti-inflammatory, antihyperalgesic, and antidepressive activity in rodents, with no evidence of toxicological signs [6]. Studies with ethyl acetate and aqueous extracts from leaves *C*. *adamantium* have demonstrated antinociceptive and anti-inflammatory effects supporting folk medicine use of these plants [7]. Phytochemical analyses have attributed the anti-inflammatory, antiproliferative and antimicrobial activities of *C*. *adamantium* to the presence of flavonoids and chalcones, which are the major constituents in the extract [5, 6, 7, 8].

The essential oil from *C*. *adamantium* leaves have been previously described as antioxidant and antimicrobial, having as major constituents in essential oil limonene, α-pinene and β-pinene [9]. Despite this evidence, there are no reports on the anti-inflammatory, antinociceptive and toxicological parameters of the essential oil of peel and seed of this fruit, which justify the present study.

Therefore, we aimed to evaluate the chemical composition of seeds and peels of essential oil from *C*. *adamantium* fruit and to their relation to acute toxicity, leukocyte migration, inflammatory and neurogenic pain and paw oedema in animal models.

## Materials and methods

### Plant material

The vegetable material collected was registered in the Authorization System and Information of Biodiversity (SISBIO), N°39462. The field studies did not involve endangered or protected species. The guavira’s fruits were produced by small farmers located at Ponta Porã-MS, Brazil, (23° 32′ 10″ S, 55° 37′ 33″ W), on November 2013.

A voucher specimen was deposited in the herbarium of the Faculty of Biological Sciences of UFGD (DDMS 4602). Fruits were sanitized and pulp, peel and seeds were separated. Peel and seeds were dried at 40°C for 62 and 72 h, respectively, protecting them from direct light until use. Humidity was calculated on a dry basis (UBS) at 70°C for 24 hours [10].

The calculation of the yield was obtained taking into account the moisture content on a dry, basis, according to the following equation:

$$\mathrm{Yield}=\frac{V_{\mathrm{oil essential}}}{W_{\mathrm{sample}}-\frac{W_{\mathrm{sample}}*H}{100}}*100$$

Where *V*_oil essential_ is oil essential volume obtained (mL), *w*_*sample*_ is initial sample mass (g) and H is the moisture content.

#### Animals

Male *Wistar* rats (200–230 g) and male and female *Swiss* mice (20–25 g) were obtained from Federal University of Grande Dourados (UFGD) biotherium. Animals were kept in collective cages—(6 animals/ cage) at a controlled temperature (23±1°C) with light cycle (12 h light/dark), drinking water and a commercial diet *ad libitum*. The 23/2014 protocol was approved by the **“**Ethics Committee on Animal Use (CEUA) of Federal University of Grande Dourados”.

### Extraction and composition of essential oil

Essential oil was obtained from 200 g of dried peel and 200 g of dried seeds from C. *adamantium* by Hydrodistillation (3 replicates) using a Clevenger-type apparatus for 3 hours. At the end of each distillation, oils were collected and transferred to glass flasks, and kept at a temperature of -18°C for further analyses. Samples obtained by hydrodistillation were analyzed by GC/qMS (Shimadzu P2010 plus. Shimadzu Tokyo, Japan) equipped with an autoinjector split/splitless. The chromatographic separation was performed on a DB-5 column (J & W. Folsom, California) 5% phenyl- dimethylpolysiloxane (30 m long × 0.25 mm diameter × 0.25 mm of film thickness) under the following conditions: carrier gas helium (99.999%) at a flow rate of 1 mL/min; 1 μL of injection volume split ratio (1:20). The temperature program of the first column started at 50°C for 5 min, heating at 3°C/min until 250°C. The injector transfer line and detector temperature used were maintained at 250°C. The MS scan parameters included electron impact ionization voltage at 70 V, a mass range from 50 to 550 Daltons and a scan interval of 0.5 s. Samples (1 mg of the essential oils) was diluted in 1 mL of n-hexane before injection.

Temperature-programmed retention indexes [11] were calculated using a mixture of normal alkanes (C_6_-C_30_) as external references. Identification of compounds was performed using retention time [12] and comparing with interpretation of mass spectra of unknown components according to Wiley mass spectra library, Wiley MS 6th Edition.

Relative peak areas for each chromatographic peak were used to evaluate the contribution of each compound to the total area and for comparisons between samples. The sum of all peak areas was considered 100% of the sample and for each peak a percentage was assigned corresponding to its area [11].

### Anti-inflammatory tests

#### Pleurisy

Different groups of female *Swiss* mice (n = 5) were orally administered with EOP (essential oil from peel) and EOS (essential oil from seed) at the doses of 100 and 300 mg/kg, respectively, in 0,9% of saline solution, which was administered as a control in a third group of animals. Positive control group received dexamethasone subcutaneously at dose of 1 mg/kg. Pleurisy was induced in the experimental groups by intrapleural injection of 100 μL of 1% carrageenan diluted in saline, after one hour of treatment (without anesthetization), as previously described [13]. Negative control received 100 μL of sterile saline by intrapleural injection. After 4 h, animals were euthanized and the pleural cavity was washed with 1 mL phosphate buffer-saline (PBS). An aliquot of 20 μL of lavage (exudate) was collected from the pleural cavity, and diluted with Turck (1:20) and used for total leukocyte count in a Neubauer chamber [14].

#### Carrageenan-induced paw oedema

Different groups of male *Swiss* mice (n = 5) were orally treated with EOP and EOS (100 and 300 mg/kg), or vehicle (control group). Another group was treated subcutaneously with dexamethasone (1 mg/kg). After 1 h, animals received a solution of 50 μL carrageenan injection (300 μg/paw) in the left hind paw. The other paw received the same volume of sterile saline 0.9%. The paw volume was measured at 1, 2, 3 and 4 hours after carrageenan injection with a pletysmometer. Results were expressed as the difference between the left and right paws at each time [4].

### Antinociceptive tests

#### Formalin-induced spontaneous pain model

Nociception was evaluated after injection of formalin [15,16]. Sixty minutes before, male *Wistar* rats (n = 5) were divided in groups: dexametasone (1mg/kg. s.c.), EOS (100 and 300 mg/kg), EOP (100 and 300 mg/kg) and vehicle (saline solution (0.9%), treated by oral route. One hour after treatments it was injected 20 μL of saline containing 2.5% of formalin in the right hind paw. Pain reaction time (paw licking) in seconds was evaluated from 0 to 5 min (phase 1—neurogenic pain) and from 15 to 30 min (phase 2—inflammatory response) after injection of formalin in the paw [14], which represents the tonic response to pain, accompanied by an inflammatory response. Following, animals were submitted to paw oedema measurement, cold sensitivity and open field tests.

#### Cold sensitivity

Cold hyperalgesia was measured by the acetone test as described by Decosterd and Woolf [17]. A needle connected to a syringe was used to drop 30 μL of acetone on the paw and the duration (in seconds) of the paw withdrawal was recorded. Minimal and maximal cut-offs were assigned at 0.5 and 20 sec, respectively. Paw withdrawals due to locomotion or weight shifting were not counted and such trials were repeated [7].

#### Open-field test

Analyses of locomotion activities were performed after formalin experiment. The rats were acclimated in locomotor measurement chambers before testing. For the test, rats were positioned in the center of an open-field apparatus, consisted of white square arena (80×80 cm) surrounded by walls (40 cm height) with its floor divided by lines into 16 squares (20×20 cm). Locomotor activity was determined by the number of squares crossed during 5 minutes. The apparatus was cleaned with 10% ethanol solution and paper towels between each section [18].

#### Formalin-induced paw oedema

Animals received an intraplantar injection of formalin (20 μL of a solution 2.5% v/v) in the right hind paw. Thickness paw oedema was assessed using a pletysmometer 30 min before any treatment and after one hour of formalin injection. Results were expressed in μL as the difference between the baseline and post-injection oedema values, with modifications of Kassuya et al. [14]

### Toxicity test

#### Acute oral toxicity

Treatments were performed by single oral administration at doses 0, 175, 560, 1792, or 2000 mg/kg of body weight of EOP and EOS. Female *Swiss* mice (n = 8) were observed for signs of toxicity during the first 0.5, 1, 2, 4, 8, 12 and 24 hours and at every 48 hours for 14 days. During the experimental period animals were observed daily for clinical aspects, including posture, seizures/tremors, consistency and appearance of the feces, eyelid closure, piloerection, appearance of skin and hair, stress, salivation, behavior, body weight and consumption of food and water [19].

### Statistical analyses

Results were expressed as mean ± standard error of the mean (S.E.M.). For comparison of results among experimental groups it was used analysis of variance (one-way ANOVA) followed by Newman-Keuls test. The number of animals per group is indicated in the legends. Statistical differences were considered significant at p<0.05.

## Results

The moisture content determined for dried peel and seed of *C*. *adamantium* were 26.07 ± 3.80% (wt, mass of water per mass of dried matter) and 7.29 ± 0.31% (wt), while the obtained essential oils yields were for peel 0.32% and seed 0.98% (w/w).

The list of compounds identified with a composition higher than 1% in oils is shown in the Table 1. The retention times, which were determined from three independent experiments, showed a coefficient of variation less than 2%. The major constituents in peel oils were limonene (13.07%) and thujopsene (6.96%) while seed oils were limonene (20.89%) and β-pinene (11.48%) (Table 1).

**Table 1:** Composition of the volatile compounds indentified in the essential oils of the peel and seed of *Campomanesia adamantium* fruits.

Compounds	R_t_	RI	LI	Composition (%)	
				Peel	Seed
Cumene	7.378	925	924	0.49	0.85
α-Pinene	7.653	932	932	4.67	8.50
Camphene	8.132	946	946	0.12	0.29
β-Pinene	9.190	976	974	4.82	11.48
Myrcene	9.667	989	988	1.07	1.85
α-Phellandrene	10.206	1003	1002	2.13	3.96
δ-Carene	10.425	1009	1008	0.19	0.11
α-Terpinene	10.679	1015	1014	0.39	0.79
o-Cymene	11.019	1023	1022	1.49	1.05
Limonene	11.186	1025	1024	13.07	20.89
1.8-Cineole	11.232	1026	1026	3.56	0.18
Z-β-Ocimene	11.449	1033	1032	0.12	-
E-β-Ocimene	12.001	1045	1044	3.24	4.12
ϒ-Terpinene	12.249	1054	1054	1.00	1.66
Terpinolene	13.724	1091	1086	1.82	2.09
Linalool	14.154	1096	1095	3.17	2.32
endo-Fenchol	14.816	1116	1114	0.11	0.21
Borneol	17.155	1168	1165	0.18	0.38
Terpinen-4-ol	17.477	1175	1174	0.79	0.89
α-Terpineol	18.101	1186	1186	2.40	2.61
Myrtenal	18.507	1196	1195	0.17	0.18
trans-Piperitol	19.007	1208	1207	0.12	0.14
trans-Carveol	19.238	1215	1215	0.12	0.13
Nerol	19.842	1228	1227	0.12	-
Carvone	20.468	1240	1239	0.12	-
Geraniol	21.020	1250	1249	0.15	-
Perilla aldehyde	22.012	1270	1269	0.13	0.10
Carvacrol	23.133	1299	1298	0.12	-
Methyl geranate	24.075	1323	1322	0.13	0.16
δ-Elemene	24.568	1335	1335	0.10	0.11
α-Ylangene	26.248	1375	1373	0.11	0.13
Isoledene	26.313	1376	1374	0.10	-
α-Copaene	26.350	1375	1374	0.97	0.94
β-Cubebene	27.006	1388	1387	0.10	-
β-Elemene	27.024	1389	1389	0.14	0.10
Sibirene	27.319	1400	1400	0.11	0.10
α-Gurjunene	27.697	1409	1409	0.33	0.33
Thujopsene	28.444	1429	1429	6.96	6.82
β-Copaene	28.497	1430	1430	0.41	0.38
Aromadendrene	29.005	1440	1439	2.78	2.15
Cedrane	29.088	1441	1441	0.23	0.13
trans-muurola-3,5-diene	29.421	1451	1451	0.10	0.10
α-Humulene	29.495	1452	1452	2.68	2.37
allo-Aromadendrene	29.830	1459	1458	0.83	0.52
trans-Cadina-1(6),4-diene	30.327	1475	1475	0.35	0.24
ϒ-Muurolene	30.528	1478	1478	1.53	1.35
Germacrene D	30.777	1485	1484	2.28	1.43
Widdra-2,4(14)-diene	30.575	1481	1481	0.69	0.53
α-Amorphene	30.660	1483	1483	0.23	0.05
β-Guaiene	31.091	1492	1492	0.35	0.16
Bicyclogermacrene	31.368	1500	1500	4.91	3.83
α-Muurolene	31.404	1501	1500	0.13	0.41
δ-Amorphene	31.903	1512	1511	0.51	0.13
ϒ-Cadinene	32.002	1513	1513	1.31	1.84
δ-Cadinene	32.292	1522	1521	3.55	3.82
Trans-Cadina-1,4-diene	32.692	1533	1533	0.23	0.27
α-Cadinene	32.910	1537	1537	0.17	0.27
Selina-3,7(11)-diene	33.216	1546	1545	0.36	0.16
Germacrene B	33.676	1560	1559	0.47	0.14
Nerolidol	33.760	1562	1561	0.40	-
Palustrol	34.072	1569	1567	0.63	0.10
Spathulenol	34.428	1578	1577	1.95	0.14
Globulol	34.993	1591	1590	4.00	-
Cubeban-11-ol s	35.163	1595	1595	1.18	0.10
Guaiol	35.365	1600	1600	0.60	0.28
Humulene epóxi II	35.637	1608	1608	1.04	0.11
Junenol	36.033	1620	1619	0.24	-
1-epi-Cubenol	36.319	1627	1627	1.00	0.23
α-acorenol	36.404	1632	1632	0.66	0.13
epi-α-Cadinol	36.750	1639	1638	0.71	0.18
α-muurolol	37.000	1645	1644	0.11	-
β-Eudesmol	37.087	1648	1649	0.69	0.56
α-Cadinol	37.285	1653	1652	0.39	0.10
Valerianol	37.437	1656	1656	0.50	0.18
Allohimachalol	37.585	1661	1661	-	0.50
7-epi-α-Eudesmol	37.603	1662	1662	1.85	-
Bulnesol	38.002	1671	1670	0.19	0.10
Eudesm-7(11)-em-4-ol	39.005	1701	1700	0.13	-

Compounds listed in order of elution from a DB-5 column. Rt, retention time (min); RI, retention indices on DB-5 capillary column; LI, literature indices.

**Fig 1:**
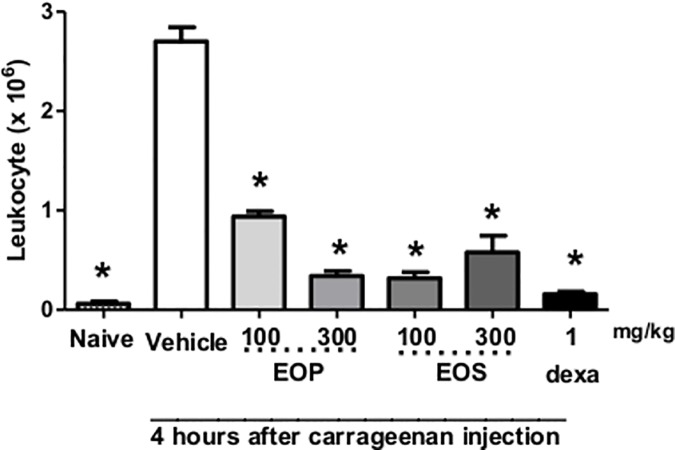
Effect of oral administration of essential oil from peel (EOP) and seed (EOS) in inhibition of leukocyte migration at both doses tested in pleurisy test. Mice were treated one hour before an intrapleural injection of carrageenan, with EOS and EOP (100 or 300 mg/kg), dexamethasone (dexa, 1 mg/kg, s.c.), or saline solution (Vehicle). Naive group, also treated with saline p.o., received an intrapleural injection of sterile saline. The bars express the mean ± SEM of 5 animals. * p<0.001 when compared to control group. Differences between groups were analyzed by analysis of variance (one-way ANOVA) followed by the Newman-Keuls test.

Oral administration of EOP and EOS significantly inhibited the leukocyte migration at all doses tested (100 and 300 mg/kg). A significant decrease was observed in leukocyte migration, at the dose of 300 mg/kg of EOP and EOS, with maximum inhibition at 89±3% for EOP and 80±6% for EOS (Fig 1).

**Fig 2:**
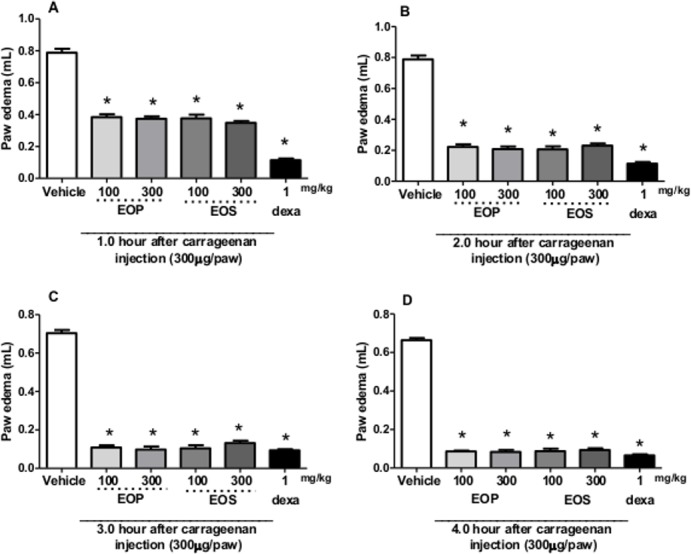
Effect of oral administration of essential oil from peel (EOP) and seed (EOS) from *Campomanesia adamantiun* in carrageenan-induced paw oedema in mice. Animals received EOP and EOS (100 or 300 mg/kg, p.o.) or control (vehicle) or dexamethasone (DEXA, 1 mg/kg, s.c.) and after 1 h an intraplantar injection of carrageenan (300 μg/paw) for evaluation of paw oedema for (A) 1, (B) 2, (C) 3, and (D) 4 h after carrageenan injection. Each bar represents the mean ± SEM of 5 animals. * p <0.001 when compared with the control group. Differences between groups were analyzed by analysis of variance (one-way ANOVA) followed by the Newman-Keuls test.

EOP and EOS caused a reduction in paw oedema induced by carrageenan (Fig 2) at all times, and maximum inhibition was at the dose of 100 mg/kg after 2 h of carrageenan injection (72±2% for EOP and 74±2% for EOS), respectively.

**Fig 3:**
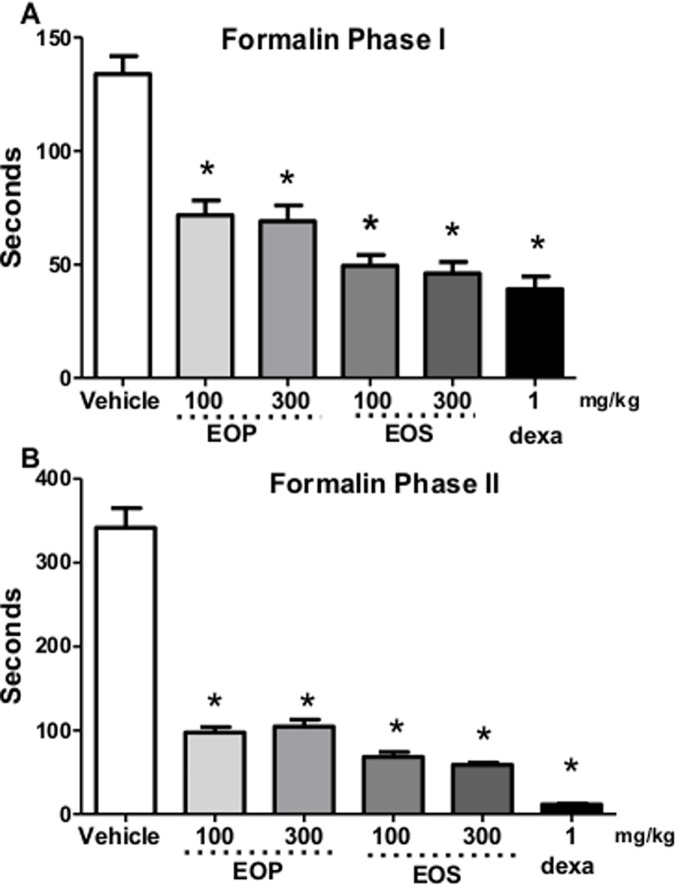
Effect of oral administration of essential oil from peel (EOP) and seed (EOS) from *C*. *adamantium* fruits in formalin-induced paw licking. Essential oil of EOS and EOP at doses of 100 and 300 mg/kg shows antinociceptive effects in phase I and II test. Results represent mean ± S.E.M. (n = 6). * *P* < 0.05, when compared to control group. Each bar represents the mean ± SEM of 5 animals. * *p* <0.001 when compared to control group. Differences among groups were analyzed by analysis of variance (one-way ANOVA) followed by the Newman-Keuls test.

EOP and EOS (Fig 3, Phase I) produced significant antinociceptive effects in the first phase when compared to control group. EOP and EOS, respectively at the doses of 100 and 300 mg/kg significantly reduced licking time in the second phase of formalin test in rats (Fig 3 Phase II). Dexametasone (1 mg/kg) produced significant antinociceptive activity in both phases of the formalin method. It was also found that orally administration of EOP and EOS inhibited formalin induced paw oedema.

**Fig 4:**
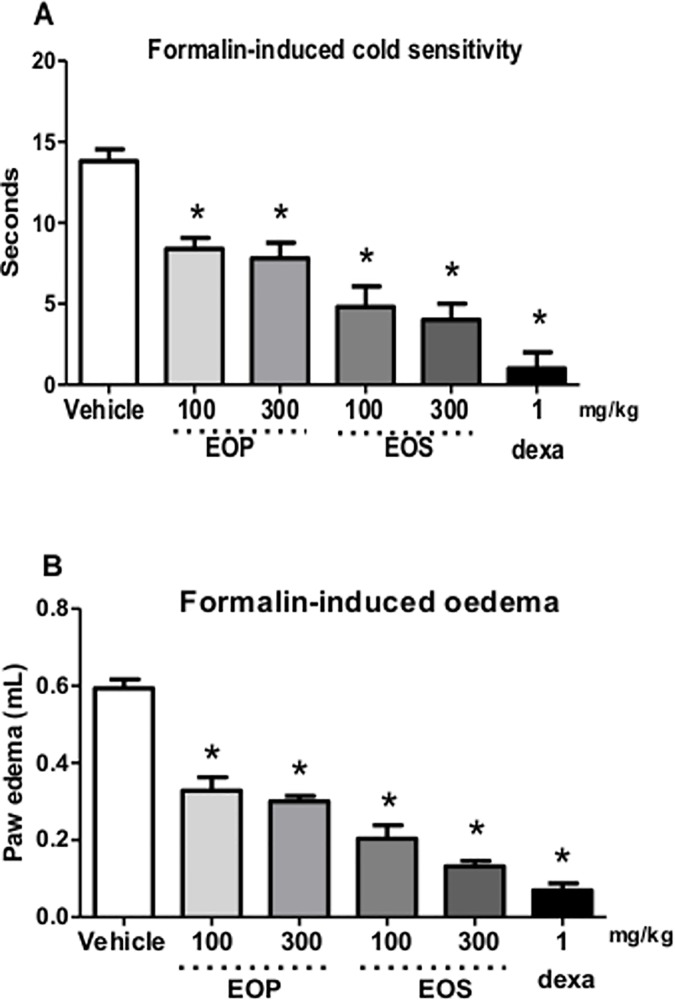
Effect of oral administration of essential oil from peel (EOP) and seed (EOS) from *C*. *adamantium* fruits in formalin-induced cold sensitivity and oedema induced by formalin. (A) Effect of essential oils of *C*. *Adamantium* fruit on cold sensitivity after treatment with EOP and EOS. (B) Oedema induced by formalin. Each bar represents the mean ± SEM of 5 animals. *P <0.001 when compared to the control group. Differences among groups were analyzed by analysis of variance (one-way ANOVA) followed by the Newman-Keuls test.

EOS administration (100 and 300 mg/kg) significantly attenuated cold hypersensitivity duration after formalin injection. Animals almost did not move and raised their paws a few times after acetone application (Fig 4A). Furthermore, hypersensitive response to cold was longer than 10 seconds (Fig 4B).

Fig 4A shows that oral treatment with the EOP and EOS with doses varying from 100 mg/kg and 300 mg/kg, the formalin-induced increased in sensitivity to a cold stimulus has significantly reduced, with a maximal inhibition at 65 ± 3%, 68 ± 2%, 75 ± 6%, 77 ± 2%, respectively, compared to the control group. Treatment with dexamethasone significantly inhibited the cold sensitivity induced by formalin at 85 ± 3% (Fig 4A).

EOP (100 mg/kg and 300 mg/kg) and EOS (100 mg/kg and 300 mg/kg) also caused a reduction in paw oedema induced by formalin. Fig 4B shows paw oedema with reduction of 49 ± 2%, 53 ± 2%, 72 ± 4%, 79 ± 3%, respectively, compared to the control group. Treatment with dexamethasone significantly inhibited the paw oedema induced by formalin by 87 ± 3% (Fig 4B).The oral treatment with essential oil from seed and peel of *C*. *adamantium* fruit did not decrease locomotor activity in open-field test (data not shown).

Toxicity of extracts and compounds were evaluated as required by regulatory agencies. Mortality is the most evident sign of toxicity. However, other aspects provide more subtle data of the adverse effects, such as loss of body mass during the study period and clinical signs of toxicity (diarrhea, piloerection and behavior changes). Animals used in the present study received essential oils (EOS and EOP) of *C*. *adamantium* and did not exhibit any clinical signs of toxicity at all doses administered and no significant changes were observed in water and food uptake. Furthermore, the absolute and relative weight of the organs (liver, kidneys and lungs) presented no statistically significant difference (Tables 2 and 3).

**Table 2:** Evaluation of weight, water consumption and feed after treatment for 14 days with the oil extracted from the peel and seed of *Campomanesia adamantium* in the acute toxicity study with mice.

Experimental group	Water consumption (mL)	Feed consumption (g)	Body weight		Weight gain (g)
			Initial weight (g)	Final weight (g)	
**Control**	104.75 ± 3.38	79.87 ± 3.59	24.16 ± 4.41	30.58 ± 5.68	6.42 ± 5.51
**EOP 175 (mg/Kg)**	103.50 ± 3.49	80.25 ± 3.97	24.75 ± 4.67	30.34 ± 3.21	5.59 ± 4.82
**EOP 560 (mg/Kg)**	100.50 ± 6.01	75.43 ± 3.67	27.53 ± 3.72	33.12 ± 4.29	5.59 ± 4.12
**EOP 1792 (mg/Kg)**	107.37 ± 3.61	89.52 ± 4.32	26.15 ± 3.52	31.97 ± 4.13	5.82 ± 4.77
**EOP 2000 (mg/Kg)**	107.25 ± 5.78	78.47 ± 5.69	25.69 ± 4.34	31.86 ± 4.11	6.17 ± 3.32
**EOS 175 (mg/Kg)**	103.42 ± 3.81	75.33 ± 3.35	23.74 ± 4.77	29.62 ± 3.96	5.88 ± 4.27
**EOS 560 (mg/Kg)**	106.82 ± 5.43	81.24 ± 3.61	24.65 ± 4.26	30.41 ± 4.52	5.76 ± 3.63
**EOS 1792 (mg/Kg)**	105.22 ± 6.77	86.88 ± 9.66	27.41 ± 4.49	33.21 ± 3.81	5.80 ± 4.76
**EOS 2000 (mg/Kg)**	108.66 ± 8.76	76.84 ± 3.61	26.39 ± 4.62	32.47 ± 5.34	6.08 ± 3.91

Values expressed as mean ± SEM. n = 8 animals/group. P< 0.05 by ANOVA.

**Table 3:** Relative weight organs of mice treated for 14 days with the oil extracted from the peel and seed of *Campomanesia adamantium* in the acute toxicity study.

Relative weight(g/100g)	Control	Bark oil 175 (mg/Kg)	Seed oil 175 (mg/Kg)	Bark oil 560 (mg/Kg)	Seed oil 560 (mg/Kg)	Bark oil 1792 (mg/Kg)	Seed oil 1792 (mg/Kg)	Bark oil 2000 (mg/Kg)	Seed oil 2000 (mg/Kg)
**Heart (g/100g)**	0.21 ± 0.08	0.20 ±0.12	0.21 ± 0.55	0.22 ± 0.01	0.20 ± 0.13	0.23 ±0.06	0.21±0.12	0.22±0.21	0.26 ±0.15
**Lung (g/100g)**	0.26 ± 0.04	0.25 ±0.18	0.28 ± 0.70	0.27 ± 0.12	0.26 ± 0.11	0.25 ±0.10	0.25±0.12	0.24± 0.16	0.25 ± 0.18
**Liver (g/100g)**	2.06 ± 0.13	1.96 ±0.14	1.80 ± 0.56	1.73 ± 0.08	1.82 ± 0.06	1.91 ±0.08	1.93±0.18	1.72± 0.14	1.98 ± 0.28
**Spleen (g/100g)**	0.19 ± 0.07	0.17 ±0.12	0.18 ± 0.55	0.12 ± 0.07	0.16 ± 0.06	0.19 ±0.07	0.14±0.42	0.15± 0.27	0.19 ± 0.21
**Right kidney (g/100g)**	0.27 ± 0.15	0.25 ±0.21	0.20 ± 0.43	0.21 ± 0.47	0.24 ± 0.44	0.23 ±0.42	0.20±0.43	0.21± 0.29	0.20 ± 0.14
**Left kidney (g/100g)**	0.25 ± 0.02	0.23 ±0.17	0.23 ± 0.73	0.22 ± 0.11	0.20 ± 0.12	0.19 ±0.11	0.22±0.02	0.19± 0.17	0.23 ± 0.73

Values expressed as mean ± SEM, n = 8 animals/group. P< 0.05 by ANOVA.

## Discussion

The present study demonstrated the anti-inflammatory, antinociceptive and toxicological analyses of EOS and EOP from *C*. *adamantium* fruits. Experimental data demonstrated that EOS and EOP inhibited leukocyte migration, inflammatory and neurogenic pain and oedema, suggesting their use as a nutraceutical or pharmacological agent.

The acute treatment with EOP and EOS did not induce changes in food and water intake or behavior (irritability, contortion, tremors, convulsions, tearing and fur). Furthermore, the absolute and relative weight of vital organs showed no statistically significant differences in any of the tested doses. Thus, the oral lethal dose (LD50) of EOP and EOS is greater than 2000 mg/kg and both can be classified as low toxicity oils according to the Organization for Economic Cooperation and Development [19]. These results corroborated with Souza et al. [6] and extended work who previously has shown that hydroalcoholic extract of *C*. *adamantium* did not exhibit toxicity in mice, as well as the essential oil.

Acute inflammation is characterized by oedema, fever, redness and pain. Oedema is an effective measure of inflammation and is useful to quantify induced cutaneous inflammation [20]. Carrageenan induced oedema is a biphasic model with multiple mediators acting in sequence to produce an inflammatory response. In the early phase, it was observed that EOP and EOS decreased carrageenan-induced paw oedema. There was significant reduction in oedema formation after 3 hours of administration of the EOP and EOS when compared with control group. Previous tests performed with *C*. *adamantium* leaves extract by Ferreira et al. [7] also demonstrated reduced oedema when compared to the control group. According to the results of this study, EOP and EOS seem to act mainly on the initial phase of the carrageenan induced inflammatory response.

Plants from the Myrtaceae Family are distributed across the Cerrado region of Brazil and some species have been used to treat pain and inflammation, among other diseases [6]. The anti-inflammatory activity of EOP and EOS in acute inflammation was assessed by carrageenan-induced pleurisy model. This is a classical test to evaluate this type of inflammation, forming an exudate in the pleural cavity characterized by infiltration of polymorphonuclear leukocytes, and the release of various chemical mediators, which are important in the inflammatory process [21].

Anti-inflammatory drugs such as indomethacin and dexamethasone are capable of inhibiting leukocyte migration between 3 and 6 h after carrageenan administration. Treatment with EOP and EOS administered 1 h before carrageenan injection was able to significantly decrease the total leukocyte recruitment into the pleural cavity. Previously work [6] has shown that hydroalcoholic extract from *C*. *adamantium* fruit barks exhibited anti-inflammatory, antihyperalgesic, and antidepressive activity in rodents. In the present work, two different parts from fruits of *C*. *adamantium* exhibited effects against the leukocyte migration induced by carrageenan in the pleura showing that the oils contain compounds with anti-inflammatory action. Formalin test is a valid and reliable model of nociception, which is predominantly used in rats and mice [22]. Pain reaction time (paw licking) in seconds, was evaluated from 0 to 5 min (phase 1 –neurogenic pain) and from 15 to 30 min (phase 2—inflammatory response) after injection of formalin in the paw, which represents the tonic response to pain, accompanied by an inflammatory response. [15,23,24].

It is accepted that the action of analgesic drugs differs in the two phases of the formalin test. While central acting drugs (opiate analgesics) inhibit both phases, anti-inflammatory drugs (non-opiate analgesics) inhibit especially the second phase [25].

EOP and EOS of *C*. *adamantium* also exhibited anti-inflammatory activity in paw oedema induced by formalin test. Therefore, the anti-inflammatory action of *C*. *adamantium* on formalin-induced paw oedema is in accordance with the results of the antinociceptive test. In the present work the essential oil from seed and peel of fruits of *C*. *adamantium* from Mato Grosso do Sul presented anti-inflammatory actions. Limonene, Thujopsene and β-pinene are the main compounds from the essential oil of *C*. *adamantium* and could be responsible for their activities.

In previous inflammatory assays, D-limonene [26, 27] exhibited a potential anti-inflammatory activity. The anti-inflammatory activity of limonene has been extensively studied, and its main mechanism involves the NFKB inhibition. [26] Limonene is one of the most common terpenes in citrus oil, constituting approximately 10% of *S*. *terebinthifolius* oil, which also demonstrated anti-inflammatory activity *in vivo* tests [18]. In other studies of essential oil from *Ocimum Kilimandscharicum* as anti-inflammatory, limonene was also found. [28].

Esteves et al. [29] have observed that Thujopsene from the essential oil of *Casearia sylvestris* leaves also presented anti-inflammatory activities. Afoulous et al. [30] demonstrated β-pinene is present in the essential oil of *Cedrelopsis* leaves and essential oils from *Citrus unshiu* flower and those have shown anti-inflammatory activities as well [31].

This is the first evaluation of the antinociceptive and anti-inflammatory effects of the essential oils of fruits (EOP and EOS) of *C*. *adamantium* in animal models. Analyzes using chromatography (GC-MS) indicated the presence of limonene, thujopsene and β-pinene that could be characterized as being contributors to the anti-inflammatory and antinociceptive effects [18, 32, 33]. Based on the literature, *C*. *adamantium* could be classified as anti-inflammatory and antinociceptive, with no evidences of toxicology.

## Conclusions

For the first time we presented the study of the essential oils of seed and peels of *C*. *adamantium* fruits, demonstrating their anti-inflammatory and antinociceptive activities. Furthermore, its usage can be considered safe because it has not caused any mortality or changes in general behavior of mice in acute toxicity studies. Further studies are needed to elucidate the mechanism of action of the oils and compounds responsible for these activities.

## Supporting information

S1 File This supporting information refers to Table 1. (PDF)

S2 File This supporting information refers to Fig 1. (PDF)

S3 File This supporting information refers to Fig 2. (PDF)

S4 File This supporting information refers to Fig 2. (PDF)

S5 File This supporting information refers to Fig 2. (PDF)

S6 File This supporting information refers to Fig 2. (PDF)

S7 File This supporting information refers to Fig 3. (PDF)

S8 File This supporting information refers to Fig 3. (PDF)

S9 File This supporting information refers to Fig 4. (PDF)

S10 File This supporting information refers to Fig 4. (PDF)
