# Supplementary material for: Seed and peel essential oils obtained from Campomanesia adamantium fruit inhibit inflammatory and pain parameters in rodents
Source: PLoS One. 2017 Feb 21;12(2):e0157107. doi: 10.1371/journal.pone.0157107 (PMC5319778; doi:10.1371/journal.pone.0157107)

Cumene

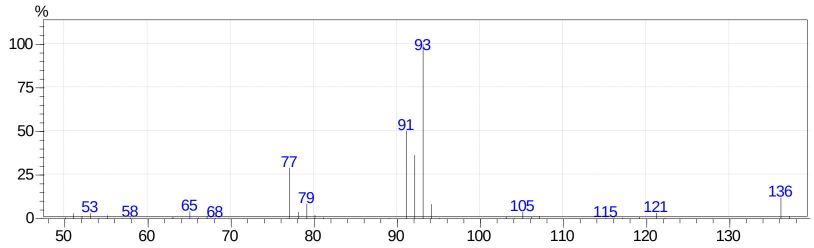

α-Pinene

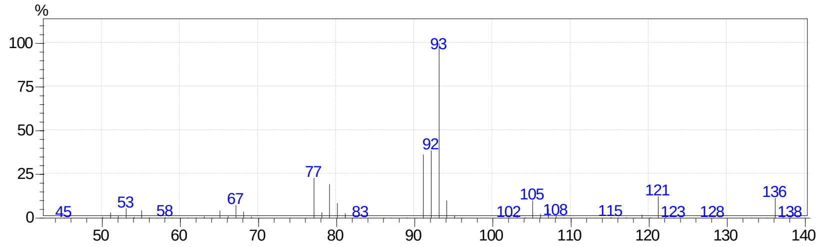

Camphene

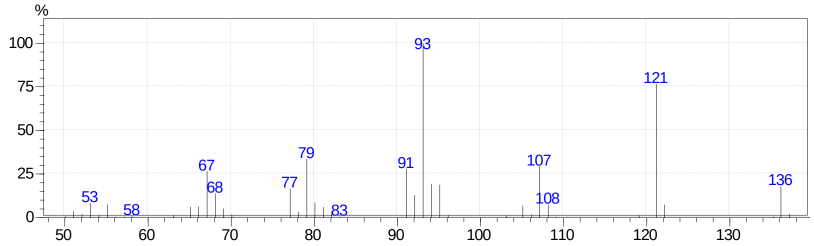

β-Pinene

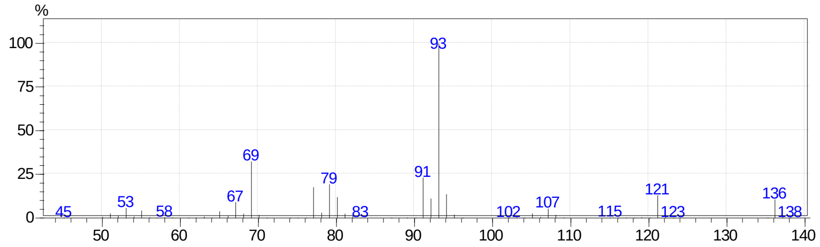

Myrcene

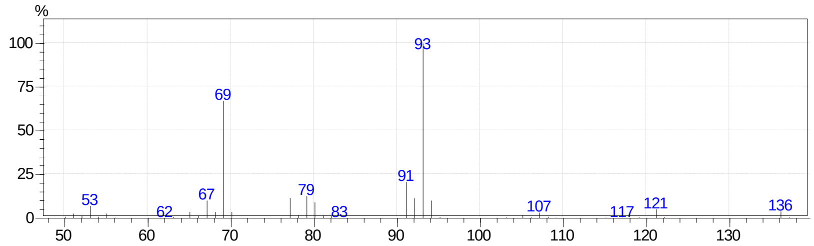

α-Phellandrene

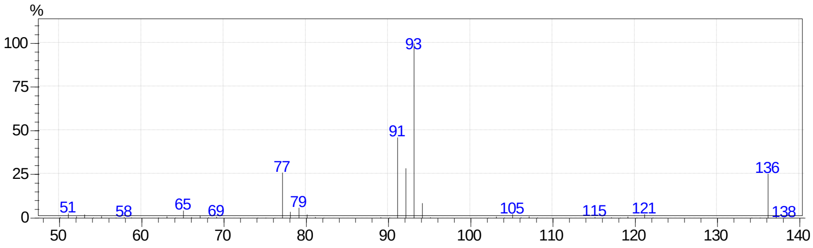

δ-Carene

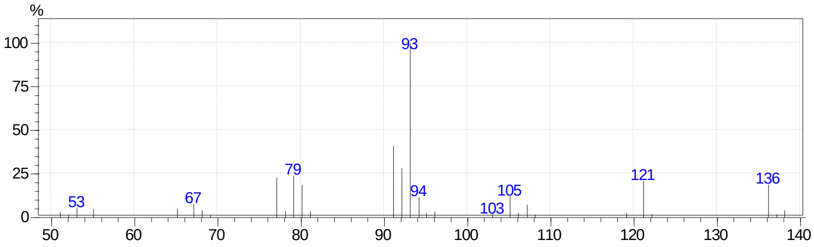

α-Terpinene

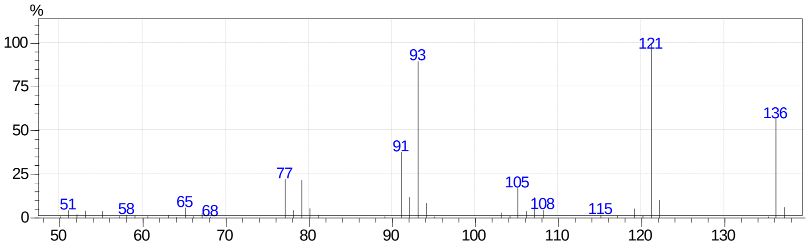

o-Cymene

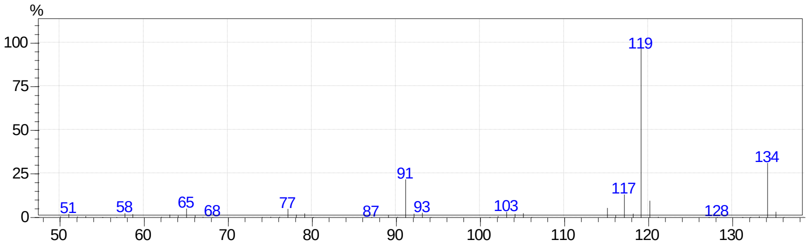

Limonene

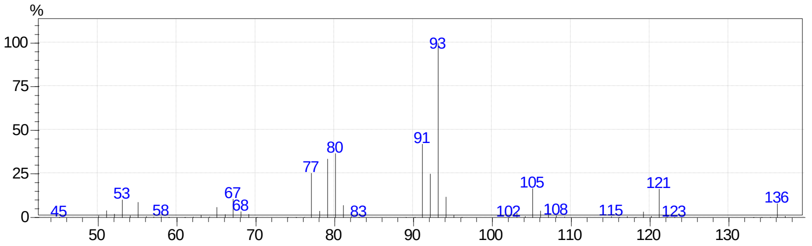

1.8-Cineole

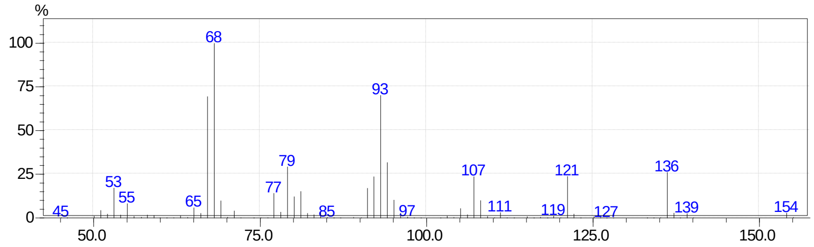

Z-β-Ocimene

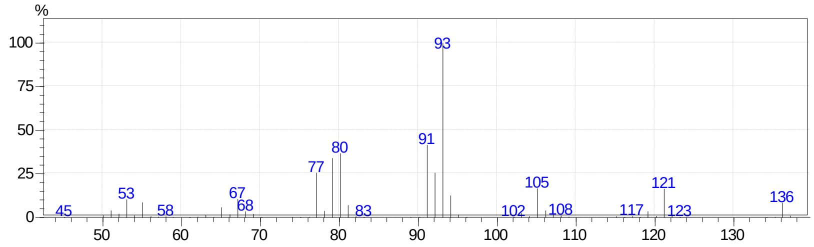

E-β-Ocimene

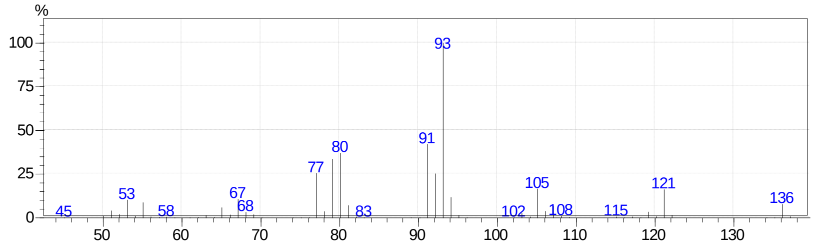

ϒ-Terpinene

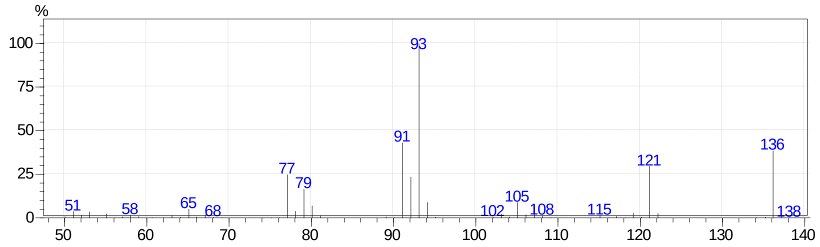

Terpinolene

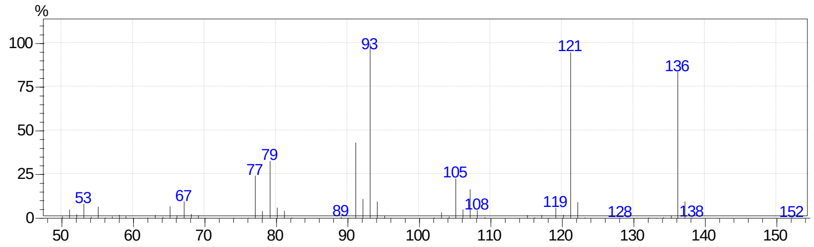

Linalool

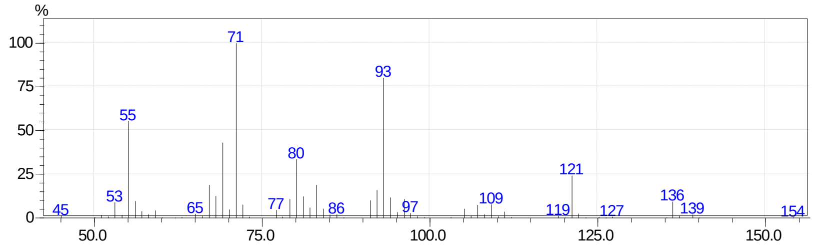

endo-Fenchol

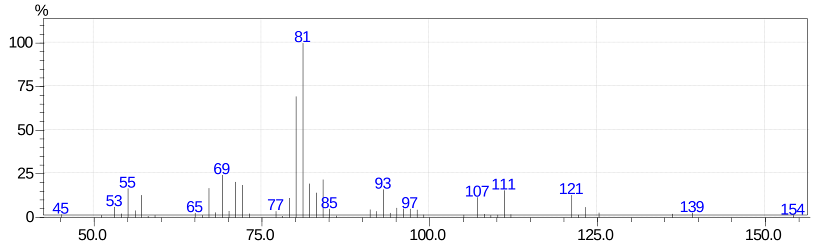

Borneol

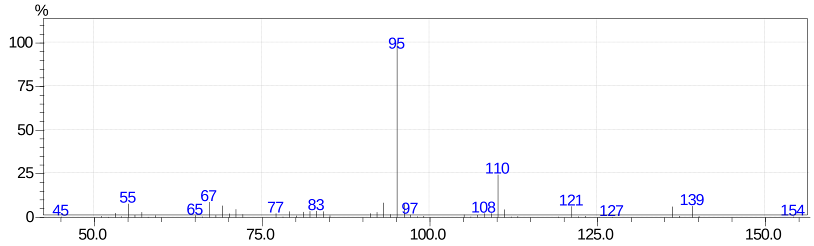

Terpinen-4-ol

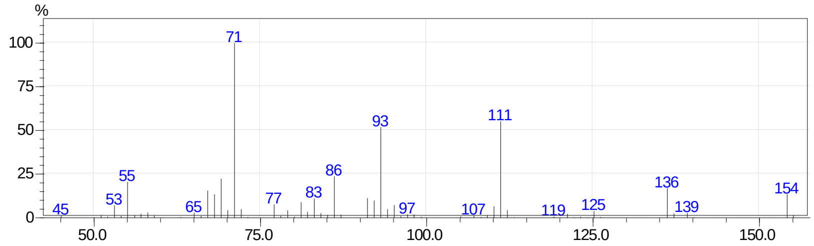

α-Terpineol

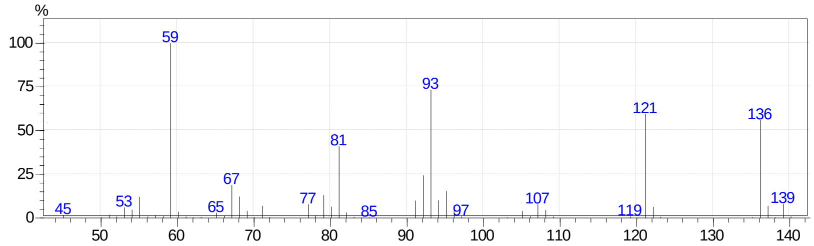

Myrtenal

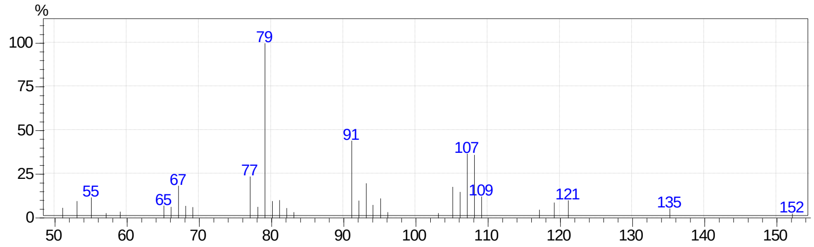

trans-Piperitol

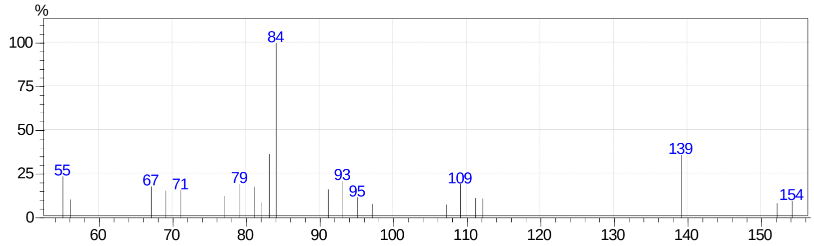

trans-Carveol

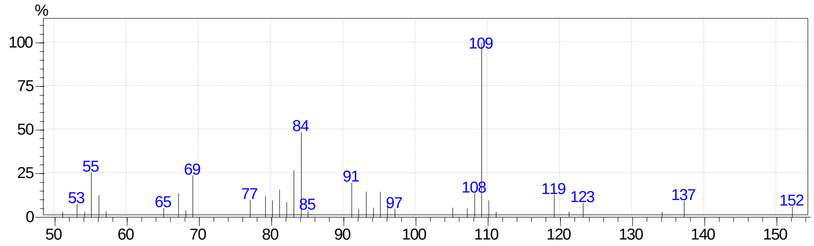

Nerol

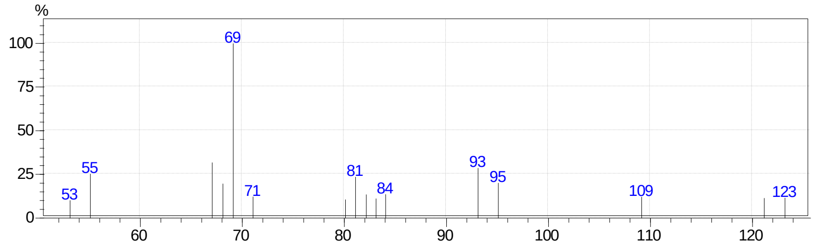

Carvone

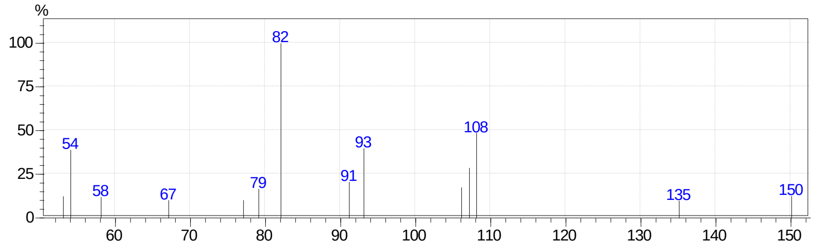

Geraniol

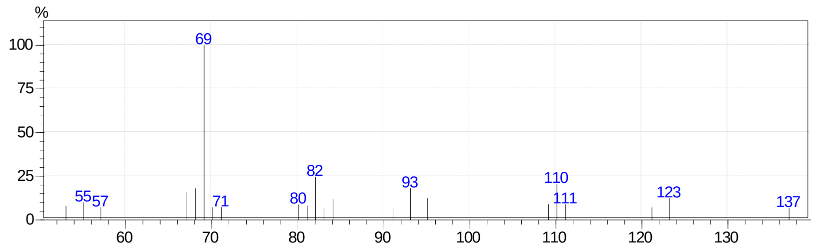

Perilla aldehyde

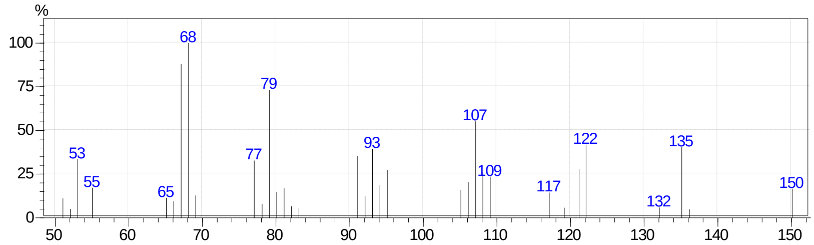

Carvacrol

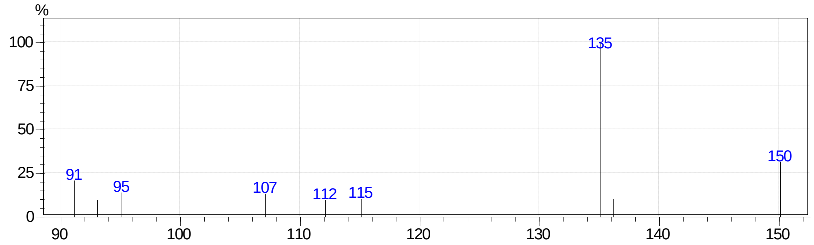

Methyl geranate

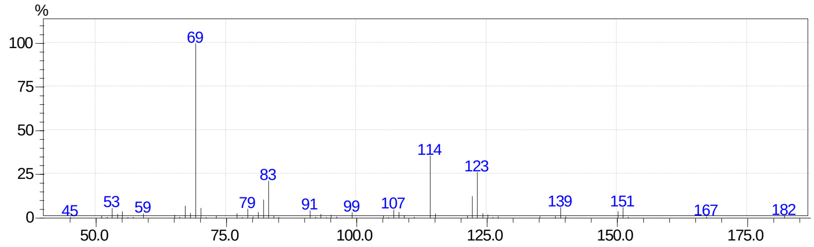

δ-Elemene

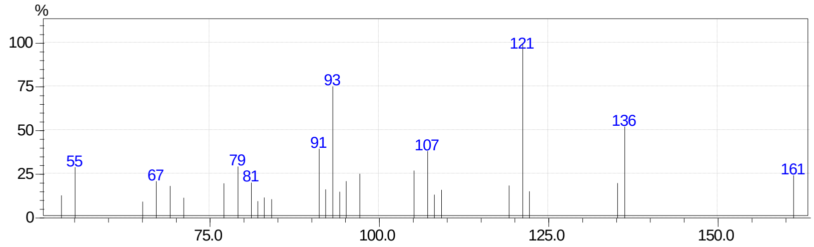

α-Ylangene

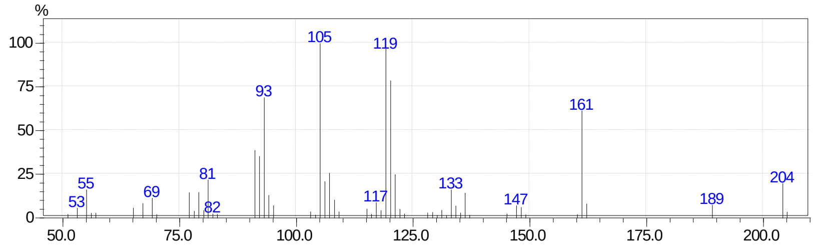

α-Copaene

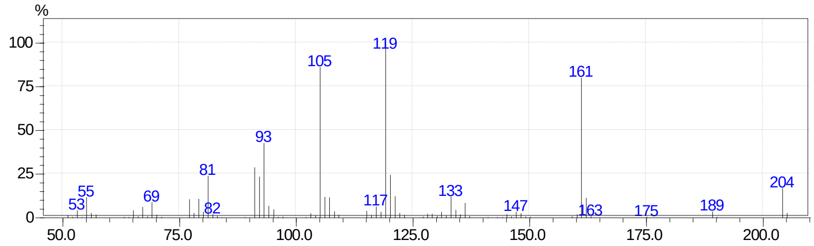

Isoledene

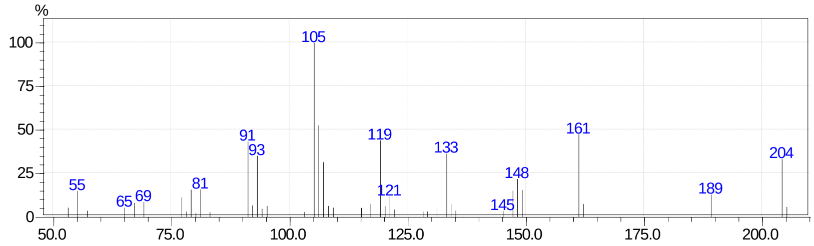

β-Cubebene

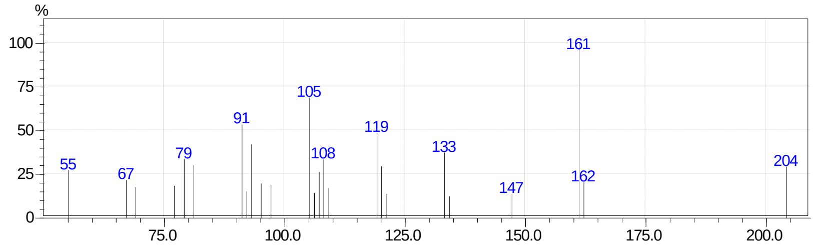

β-Elemene

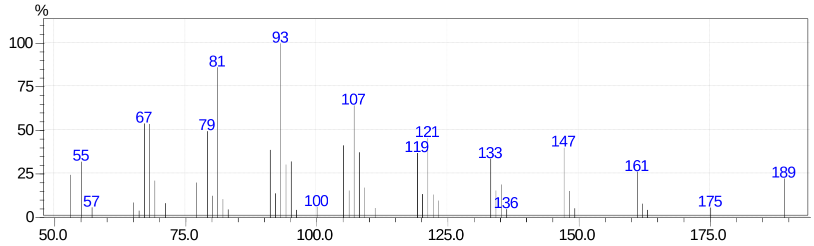

Sibirene

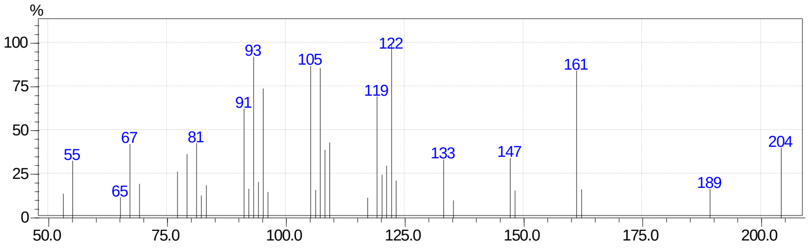

α-Gurjunene

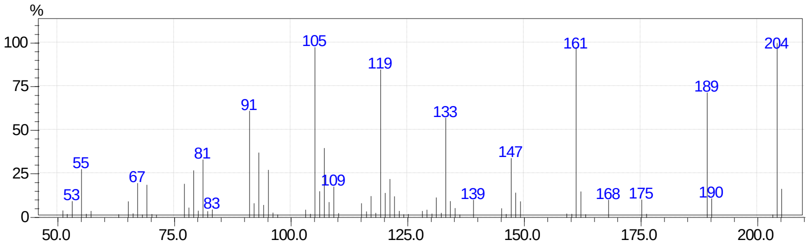

Thujopsene

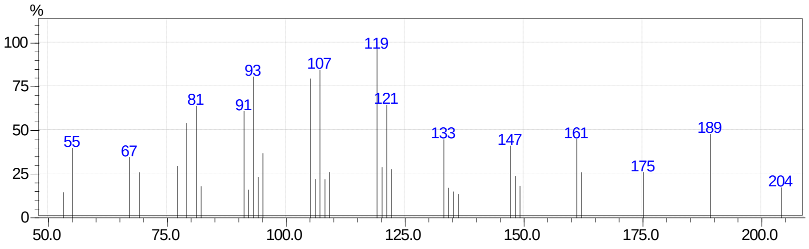

β-Copaene

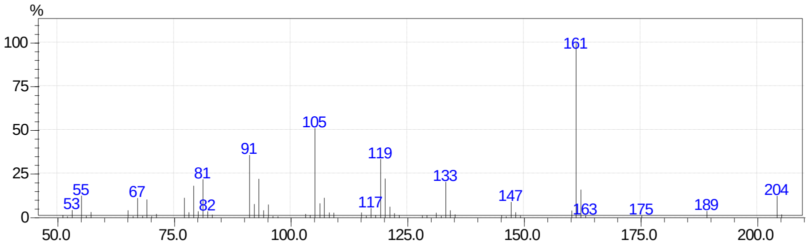

Aromadendrene

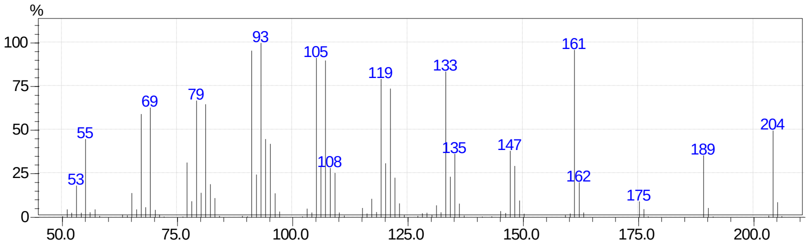

trans-muurola-3,5-diene

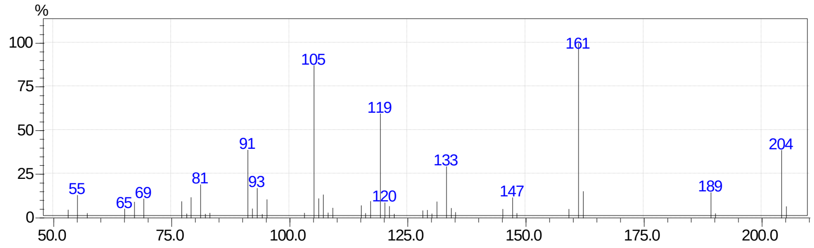

α-Humulene

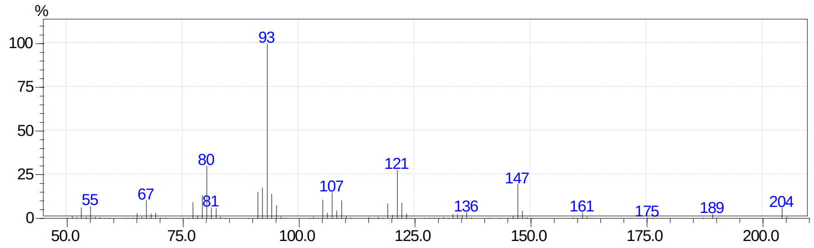

allo-Aromadendrene

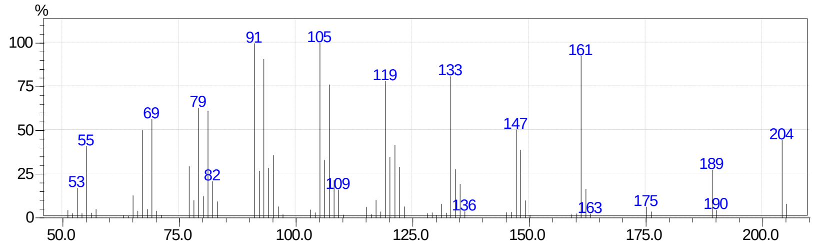

Cedrane

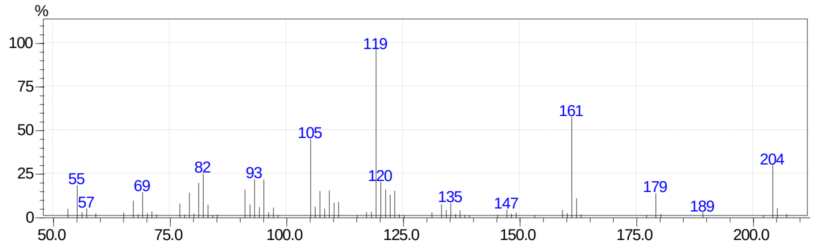

trans-Cadina-1(6),4-diene

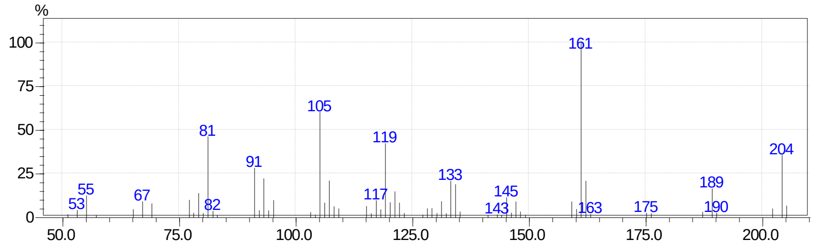

ϒ-Muurolene

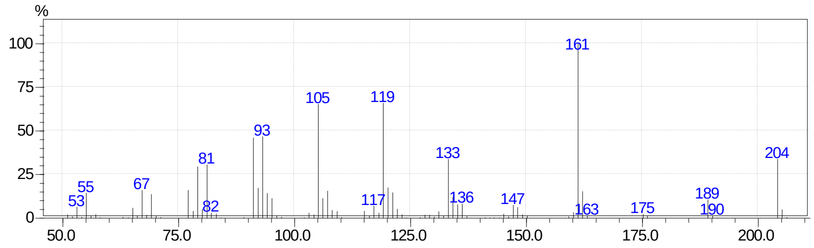

Germacrene D

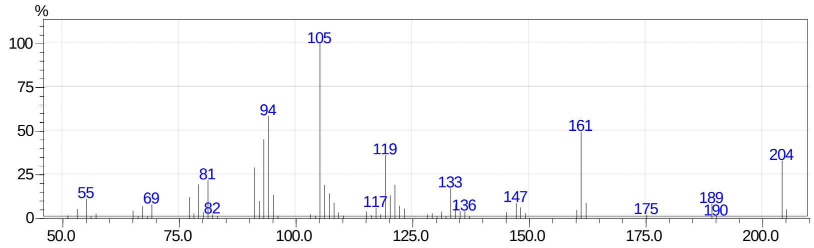

Widdra-2,4(14)-diene

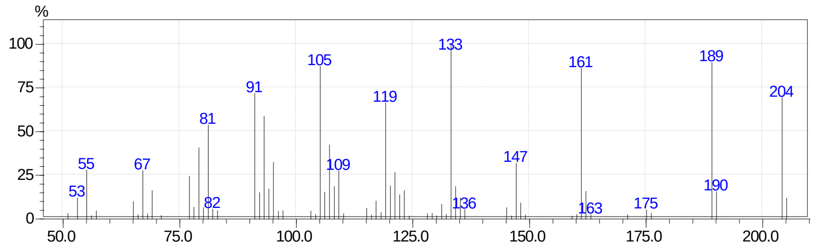

α-Amorphene

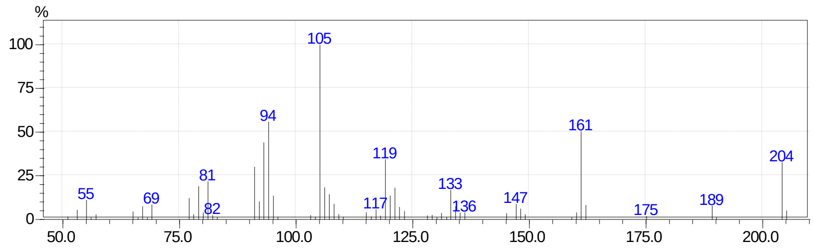

β-Guaiene

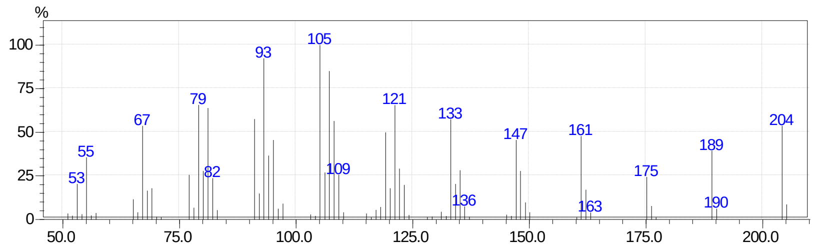

Bicyclogermacrene

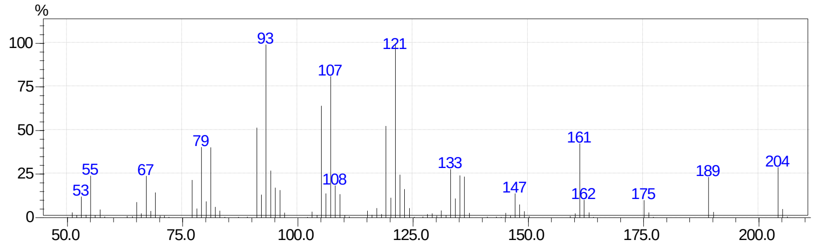

α-Muurolene

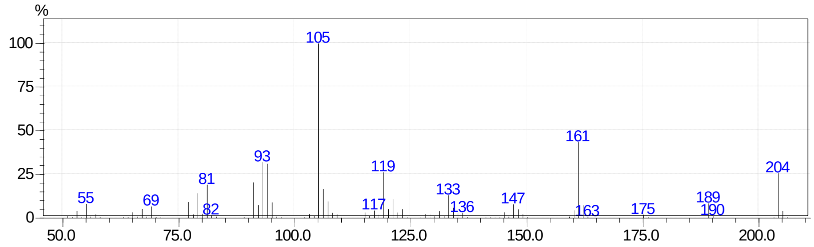

δ-Amorphene

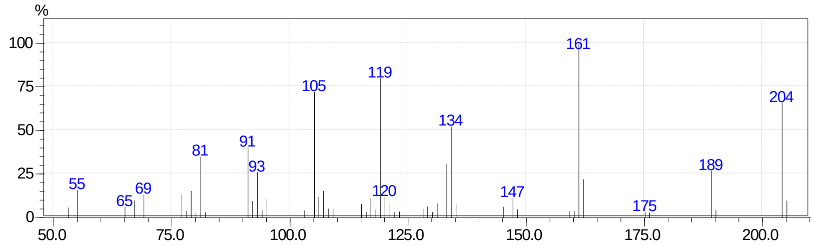

ϒ-Cadinene

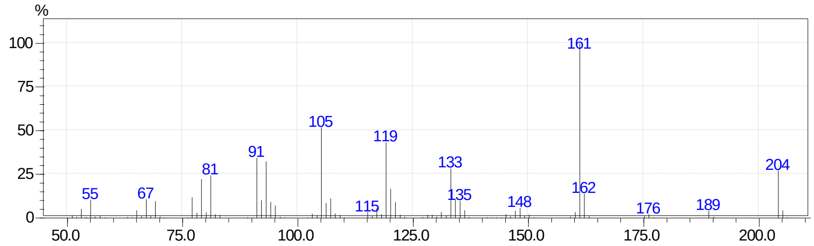

δ-Cadinene

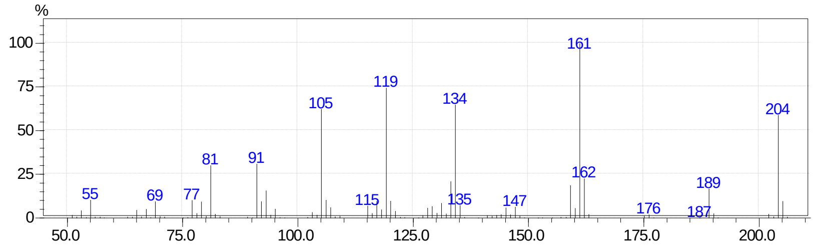

Trans-Cadina-1,4-diene

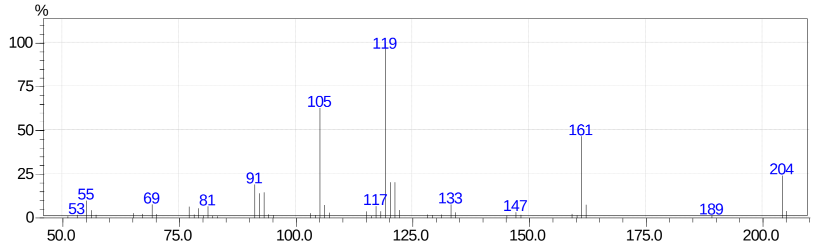

α-Cadinene

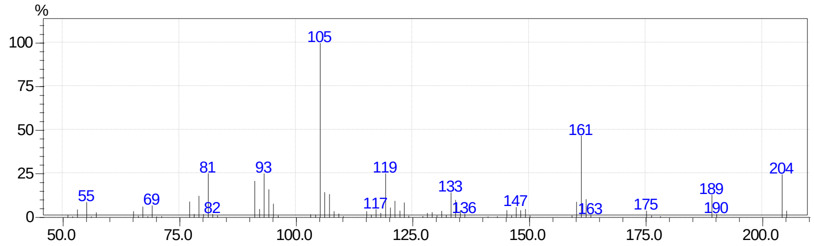

Selina-3,7(11)-diene

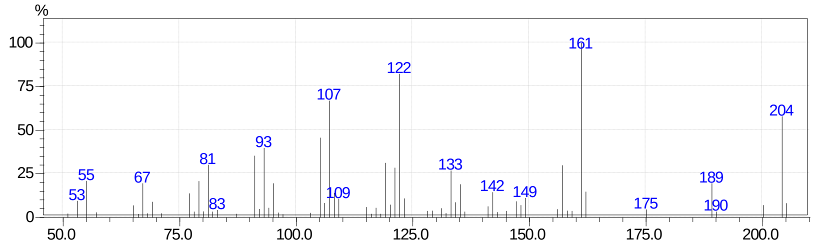

Germacrene B

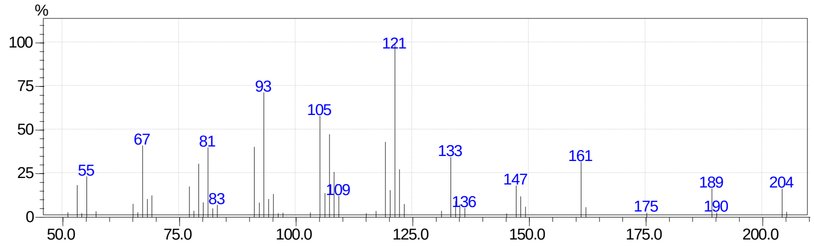

Nerolidol

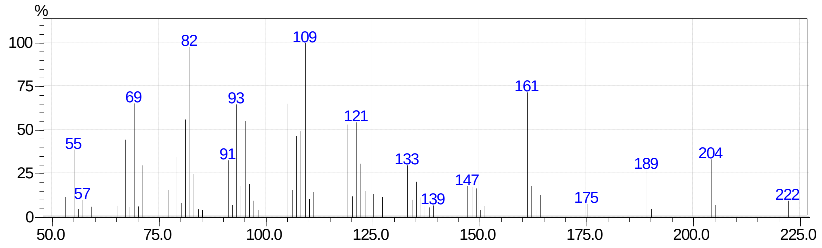

Palustrol

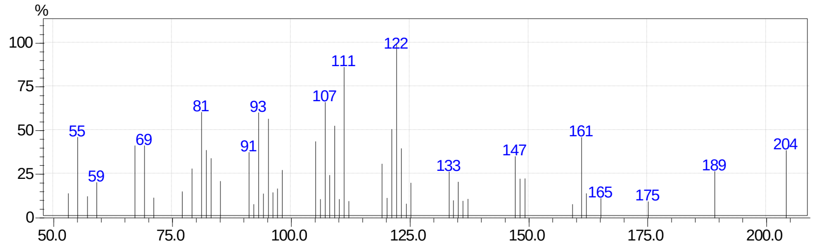

Spathulenol

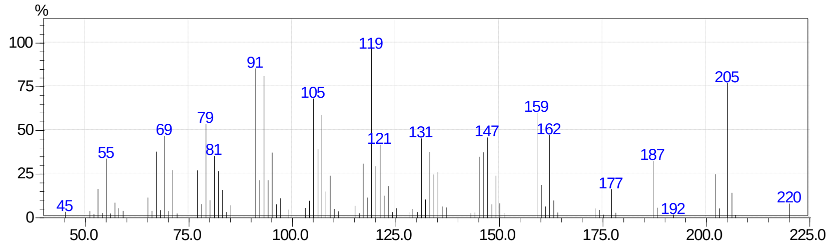

Globulol

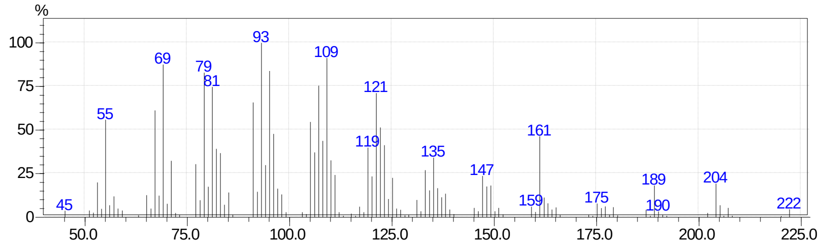

Cubeban-11-ol

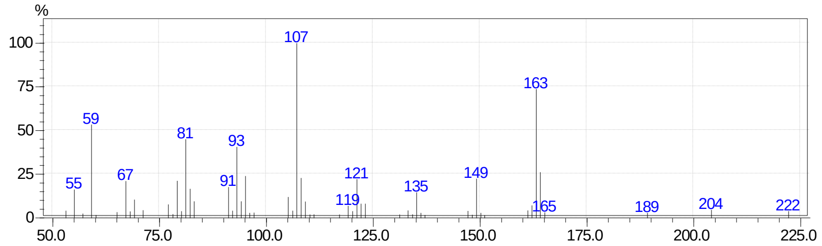

Guaiol

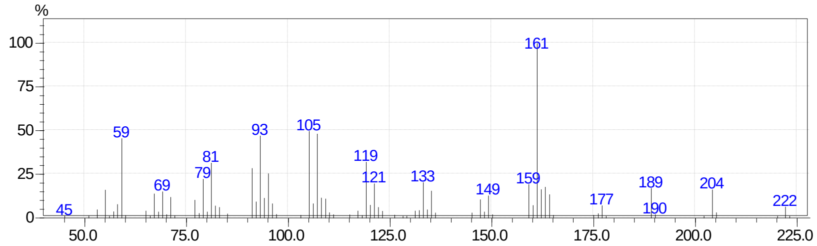

α-Atlantol (humulene epóxi II)

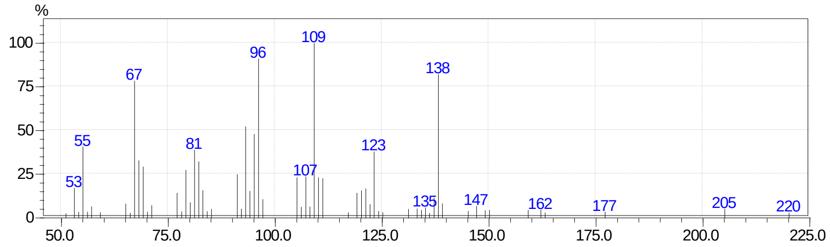

Junenol

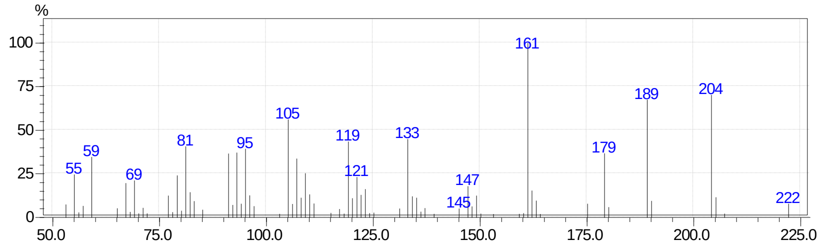

1-epi-Cubenol

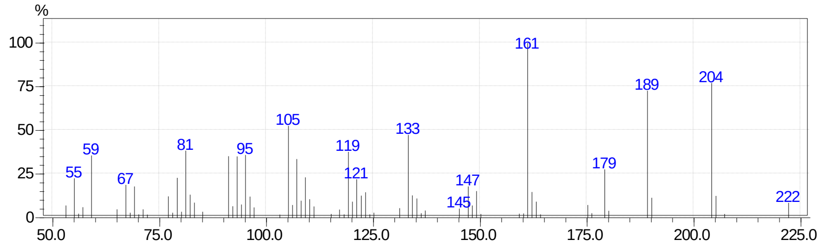

α-acorenol

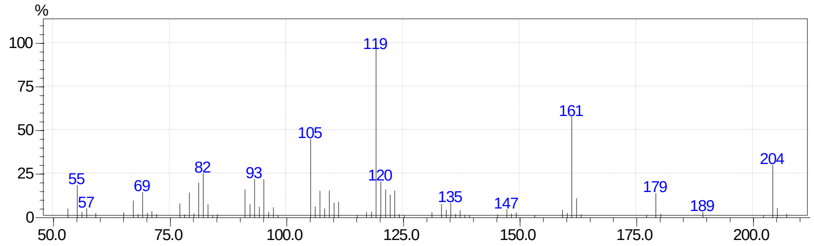

epi-α-Cadinol

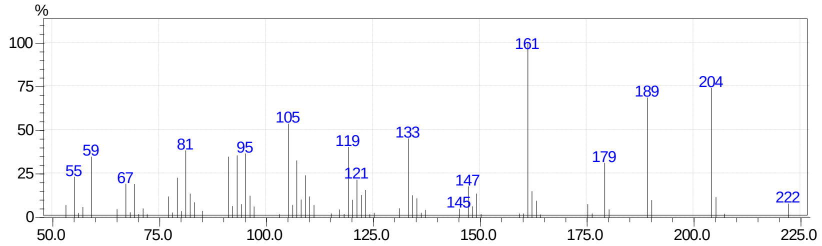

α-muurolol

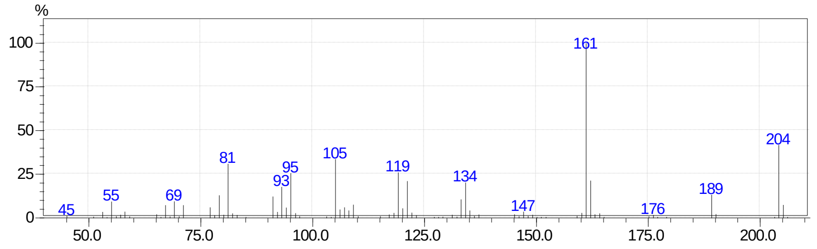

β-Eudesmol

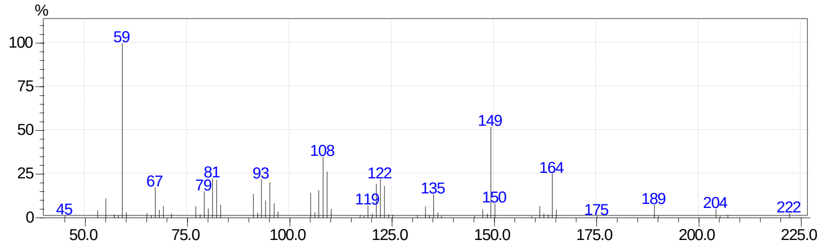

α-Cadinol

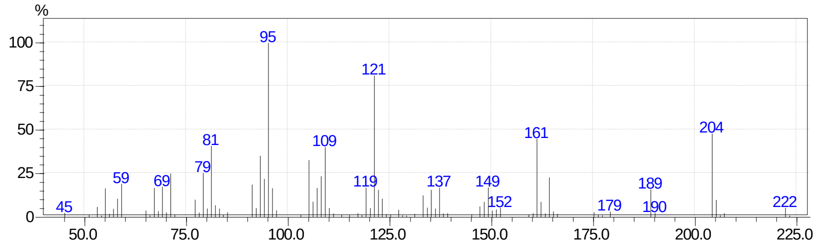

Valerianol

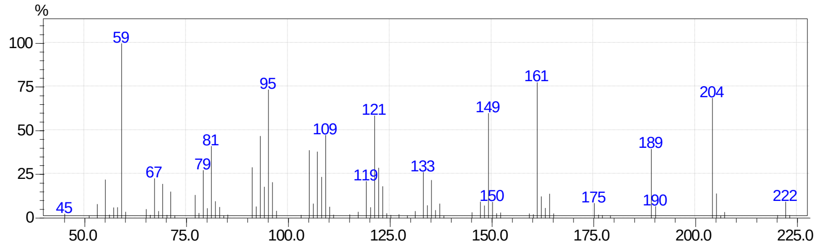

Allohimachalol

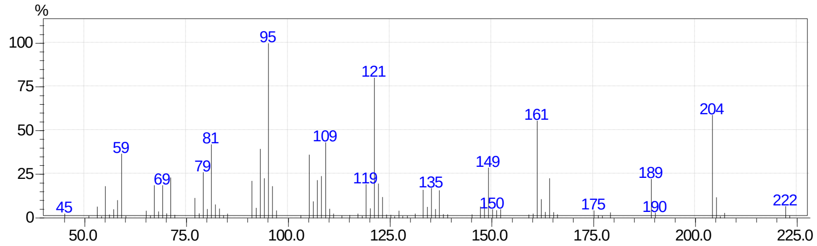

7-epi-α-Eudesmol

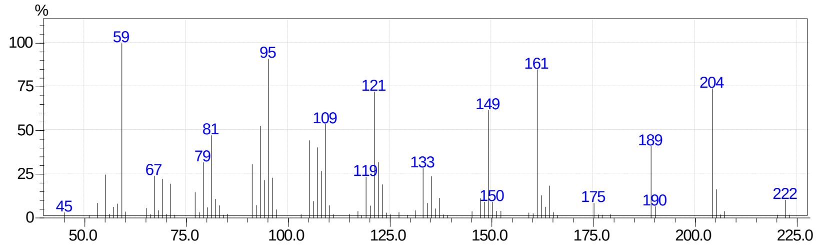

Bulnesol

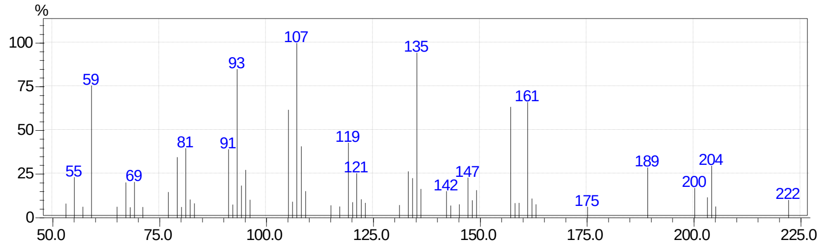

Eudesm-7(11)-em-4-ol

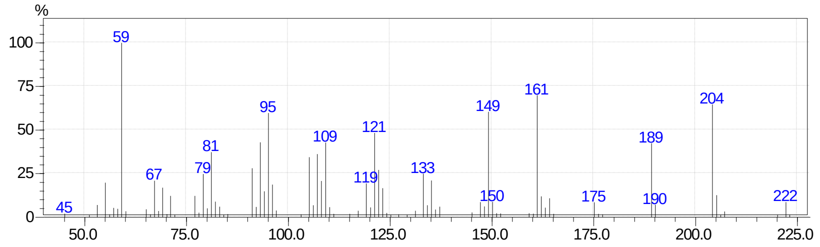

Supplement: S1 File — (PDF) [file pone.0157107.s001.pdf]
