# Supplementary material for: Seed and peel essential oils obtained from Campomanesia adamantium fruit inhibit inflammatory and pain parameters in rodents
Source: PLoS One. 2017 Feb 21;12(2):e0157107. doi: 10.1371/journal.pone.0157107 (PMC5319778; doi:10.1371/journal.pone.0157107)
Supplement: S2 File — (PDF) [file pone.0157107.s002.pdf]

alFile_Manuscript_Danieli_Microcapsules_27.07.16-marked-copy.doc]

hange Arrange Window Help

Undo | Clipboard | Analysis | Change | Import | Draw | Write | Text | Export | Print | Send | Help

Analyze | Open Prism Help | Enter search terms

	A	B	C	D	E	F	G	H	I	J	K	L
	Data Set-A	Data Set-B	Data Set-C	Data Set-D	Data Set-E	Title	Title	Title	Title	Title	Title	Title
	Y	Y	Y	Y	Y	Y	Y	Y	Y	Y	Y	Y
1	0.10	1.93	1.10	1.18	0.1							
2	0.00	1.74	1.23	1.10	0.2							
3	0.10	1.82	1.16	0.92	0.1							
4	0.00	2.21	1.14	0.84	0.2							
5	0.10	2.10	1.20	0.86	0.2							
6												
7												
8												
9												
10												
11												
12												
13												
14												
15												
16												
17												
18												
19												
20												
21												
22												
23												
24												

One-way ANOVA data | Row CT, Column A | Selected: Rows 1, Columns 5

PT
