# Supplementary material for: Seed and peel essential oils obtained from Campomanesia adamantium fruit inhibit inflammatory and pain parameters in rodents
Source: PLoS One. 2017 Feb 21;12(2):e0157107. doi: 10.1371/journal.pone.0157107 (PMC5319778; doi:10.1371/journal.pone.0157107)
Supplement: S3 File — (PDF) [file pone.0157107.s003.pdf]

\Users\Ana Claudia\Desktop\FinalFile_Manuscript_Danieli_Microcapsules_27.07.16-marked-copy.doc]

hange Arrange Window Help

Undo | Clipboard | Analysis | Change | Import | Draw | Write | Text | Export | Print | Send | Help

Analyze | Open Prism Help | Enter search terms

	A	B	C	D	E	F	G	H	I	J
	Data Set-A	Data Set-B	Data Set-C	Data Set-D	Title	Title	Title	Title	Title	Title
	Y	Y	Y	Y	Y	Y	Y	Y	Y	Y
1	0.72	0.5600	0.56	0.11						
2	0.75	0.6400	0.54	0.12						
3	0.86	0.7800	0.68	0.15						
4	0.79	0.7400	0.65	0.10						
5	0.82	0.6800	0.76	0.09						
6										
7										
8										
9										
10										
11										
12										
13										
14										
15										
16										
17										
18										
19										
20										
21										
22										
23										
24										

One-way ANOVA data | Row CT, Column A | Selected: Rows 1, Columns 4

PT
