# Supplementary material for: Seed and peel essential oils obtained from Campomanesia adamantium fruit inhibit inflammatory and pain parameters in rodents
Source: PLoS One. 2017 Feb 21;12(2):e0157107. doi: 10.1371/journal.pone.0157107 (PMC5319778; doi:10.1371/journal.pone.0157107)
Supplement: S4 File — (PDF) [file pone.0157107.s004.pdf]

\Users\Ana Claudia\Desktop\FinalFile_Manuscript_Danieli_Microcapsules_27.07.16-marked-copy.doc]

	A	B	C	D
	Data Set-A	Data Set-B	Data Set-C	Data Set-D
	Y	Y	Y	Y
1	0.72	0.35	0.360	0.11
2	0.75	0.39	0.370	0.12
3	0.86	0.40	0.390	0.15
4	0.79	0.34	0.330	0.10
5	0.82	0.44	0.420	0.09

One-way ANOVA data

Row CT, Column A

Selected: Rows 1, Columns 4
