# Supplementary material for: Seed and peel essential oils obtained from Campomanesia adamantium fruit inhibit inflammatory and pain parameters in rodents
Source: PLoS One. 2017 Feb 21;12(2):e0157107. doi: 10.1371/journal.pone.0157107 (PMC5319778; doi:10.1371/journal.pone.0157107)
Supplement: S5 File — (PDF) [file pone.0157107.s005.pdf]

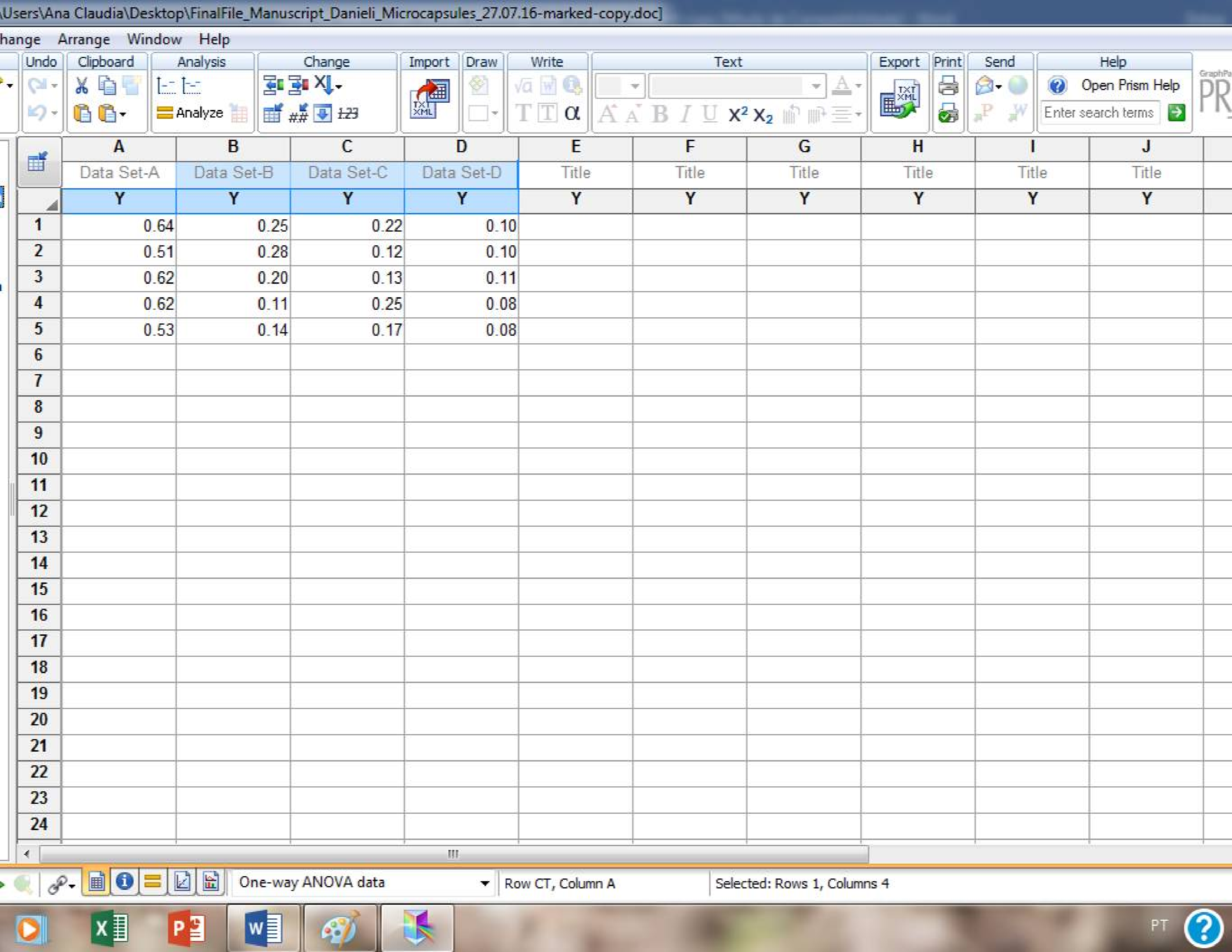
\Users\Ana Claudia\Desktop\FinalFile_Manuscript_Danieli_Microcapsules_27.07.16-marked-copy.doc]
hange Arrange Window Help
Undo Clipboard Analysis Change Import Draw Write Text Export Print Send Help
Analyze
Open Prism Help
Enter search terms
A B C D E F G H I J
Data Set-A Data Set-B Data Set-C Data Set-D Title Title Title Title Title Title
Y Y Y Y Y Y Y Y Y Y
1 0.64 0.25 0.22 0.10
2 0.51 0.28 0.12 0.10
3 0.62 0.20 0.13 0.11
4 0.62 0.11 0.25 0.08
5 0.53 0.14 0.17 0.08
One-way ANOVA data
Row CT, Column A
Selected: Rows 1, Columns 4
PT
