# Supplementary material for: Seed and peel essential oils obtained from Campomanesia adamantium fruit inhibit inflammatory and pain parameters in rodents
Source: PLoS One. 2017 Feb 21;12(2):e0157107. doi: 10.1371/journal.pone.0157107 (PMC5319778; doi:10.1371/journal.pone.0157107)
Supplement: S6 File — (PDF) [file pone.0157107.s006.pdf]

\Users\Ana Claudia\Desktop\FinalFile_Manuscript_Danieli_Microcapsules_27.07.16-marked-copy.doc]

hange Arrange Window Help

Undo | Clipboard | Analysis | Change | Import | Draw | Write | Text | Export | Print | Send | Help

Analyze

Open Prism Help

Enter search terms

	A	B	C	D	E	F	G	H	I	J	K	L
	Data Set-A	Data Set-B	Data Set-C	Data Set-D	Title	Title	Title	Title	Title	Title	Title	Title
	Y	Y	Y	Y	Y	Y	Y	Y	Y	Y	Y	Y
1	0.64	0.35	0.32	0.10								
2	0.51	0.38	0.22	0.10								
3	0.62	0.30	0.23	0.11								
4	0.62	0.21	0.20	0.08								
5	0.53	0.34	0.20	0.08								
6												
7												
8												
9												
10												
11												
12												
13												
14												
15												
16												
17												
18												
19												
20												
21												
22												
23												
24												

One-way ANOVA data | Row CT, Column A | Selected: Rows 1, Columns 4

PT
