# Supplementary material for: Seed and peel essential oils obtained from Campomanesia adamantium fruit inhibit inflammatory and pain parameters in rodents
Source: PLoS One. 2017 Feb 21;12(2):e0157107. doi: 10.1371/journal.pone.0157107 (PMC5319778; doi:10.1371/journal.pone.0157107)
Supplement: S8 File — (PDF) [file pone.0157107.s008.pdf]

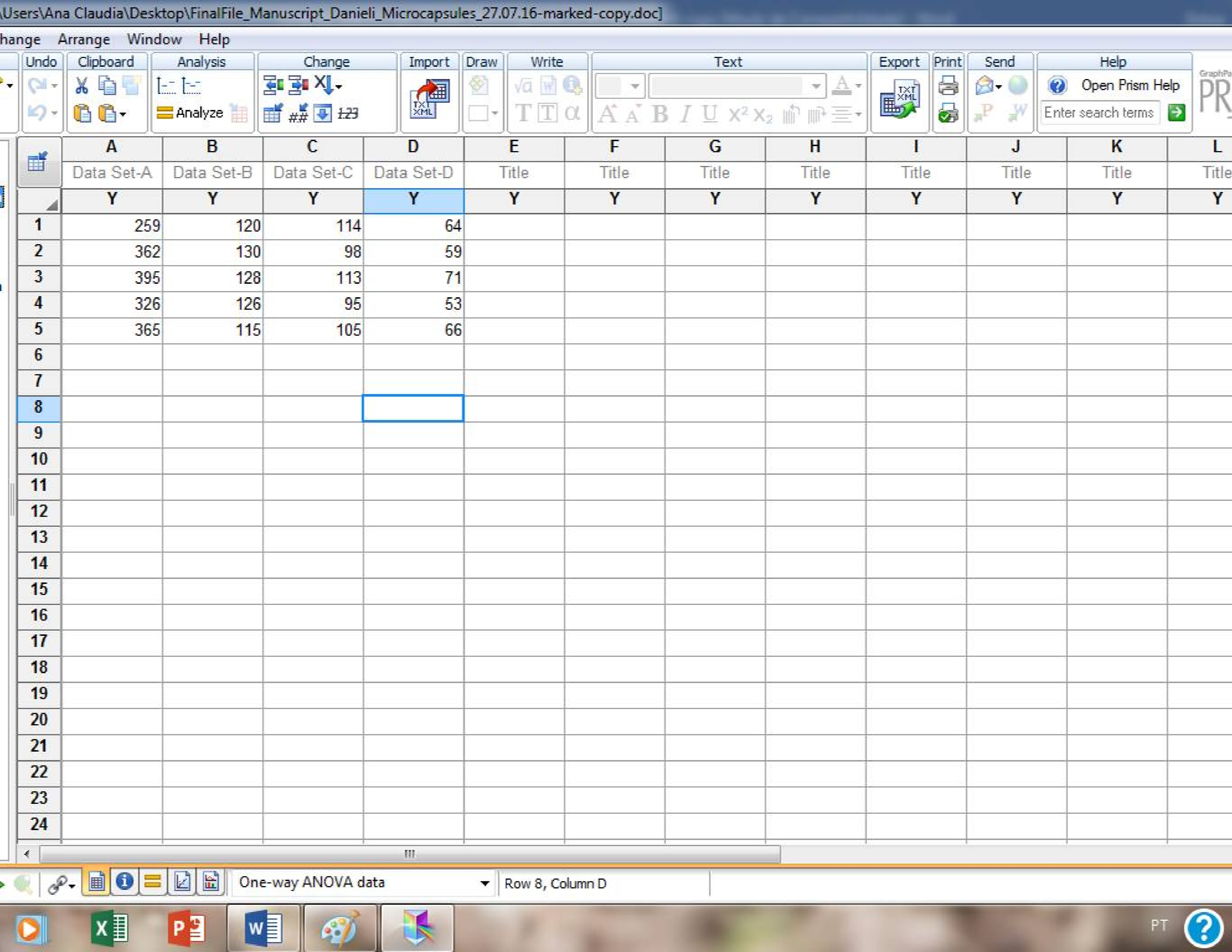
\Users\Ana Claudia\Desktop\FinalFile_Manuscript_Danieli_Microcapsules_27.07.16-marked-copy.doc]
hange Arrange Window Help
Undo Clipboard Analysis Change Import Draw Write Text Export Print Send Help
Analyze
Open Prism Help
Enter search terms
A B C D E F G H I J K L
Data Set-A Data Set-B Data Set-C Data Set-D Title Title Title Title Title Title Title Title
Y Y Y Y Y Y Y Y Y Y Y Y
1 259 120 114 64
2 362 130 98 59
3 395 128 113 71
4 326 126 95 53
5 365 115 105 66
One-way ANOVA data
Row 8, Column D
PT
