# Supplementary material for: Seed and peel essential oils obtained from Campomanesia adamantium fruit inhibit inflammatory and pain parameters in rodents
Source: PLoS One. 2017 Feb 21;12(2):e0157107. doi: 10.1371/journal.pone.0157107 (PMC5319778; doi:10.1371/journal.pone.0157107)
Supplement: S9 File — (PDF) [file pone.0157107.s009.pdf]

\Users\Ana Claudia\Desktop\FinalFile_Manuscript_Danieli_Microcapsules_27.07.16-marked-copy.doc]

hange Arrange Window Help

Undo | Clipboard | Analysis | Change | Import | Draw | Write | Text | Export | Print | Send | Help

Analyze | Open Prism Help | Enter search terms

| | A | B | C | D | E | F | G | H | I | J |
|---|---|---|---|---|---|---|---|---|---|---|
| | Data Set-A | Data Set-B | Data Set-C | Data Set-D | Title | Title | Title | Title | Title | Title |
| | Y | Y | Y | Y | Y | Y | Y | Y | Y | Y |
| 1 | 12.00 | 7.00 | 8.00 | 0.00 | | | | | | |
| 2 | 15.00 | 10.00 | 9.00 | 0.00 | | | | | | |
| 3 | 12.00 | 10.00 | 8.00 | 0.00 | | | | | | |
| 4 | 15.00 | 8.00 | 7.00 | 5.00 | | | | | | |
| 5 | 15.00 | 7.00 | 9.00 | 0.00 | | | | | | |
| 6 | | | | | | | | | | |
| 7 | | | | | | | | | | |
| 8 | | | | | | | | | | |
| 9 | | | | | | | | | | |
| 10 | | | | | | | | | | |
| 11 | | | | | | | | | | |
| 12 | | | | | | | | | | |
| 13 | | | | | | | | | | |
| 14 | | | | | | | | | | |
| 15 | | | | | | | | | | |
| 16 | | | | | | | | | | |
| 17 | | | | | | | | | | |
| 18 | | | | | | | | | | |
| 19 | | | | | | | | | | |
| 20 | | | | | | | | | | |
| 21 | | | | | | | | | | |
| 22 | | | | | | | | | | |
| 23 | | | | | | | | | | |
| 24 | | | | | | | | | | |

One-way ANOVA data | Row 15, Column C

PT
