# Supplementary material for: Seed and peel essential oils obtained from Campomanesia adamantium fruit inhibit inflammatory and pain parameters in rodents
Source: PLoS One. 2017 Feb 21;12(2):e0157107. doi: 10.1371/journal.pone.0157107 (PMC5319778; doi:10.1371/journal.pone.0157107)
Supplement: S10 File — (PDF) [file pone.0157107.s010.pdf]

\Users\Ana Claudia\Desktop\FinalFile_Manuscript_Danieli_Microcapsules_27.07.16-marked-copy.doc]

A	B	C	D
Data Set-A	Data Set-B	Data Set-C	Data Set-D
Y	Y	Y	Y
0.60	0.42	0.25	0.02
0.57	0.32	0.30	0.10
0.52	0.45	0.29	0.06
0.63	0.38	0.24	0.12
0.65	0.28	0.26	0.05

One-way ANOVA data

Row 4, Column C
